# Efficient Microwave Irradiation-Assisted Synthesis of Benzodioxinoquinoxaline and Its Donor-Variegated Derivatives Enabling Long-Lived Emission and Efficient Bipolar Charge Carrier Transport

**DOI:** 10.1021/acsmaterialsau.4c00050

**Published:** 2024-08-14

**Authors:** Liliia Deva, Mariia Stanitska, Levani Skhirtladze, Amjad Ali, Glib Baryshnikov, Dmytro Volyniuk, Stepan Kutsiy, Mykola Obushak, Monika Cekaviciute, Pavlo Stakhira, Juozas Vidas Grazulevicius

**Affiliations:** † Department of Electronic Engineering, Institute of Telecommunications, Radioelectronics and Electronic Engineering, Lviv Polytechnic National University, Stepan Bandera st. 12, Lviv 79013, Ukraine; ‡ Kaunas University of Technology, Baršausko 59, Kaunas 51423, Lithuania; § Ivan Franko National University of Lviv, Kyryla i Mefodiya 6, Lviv 79000, Ukraine; ∥ Laboratory of Organic Electronics, Department of Science and Technology, Linköping University, Norrköping SE-60174, Sweden

**Keywords:** microwave irradiation-assisted Buchwald−Hartwig cross-coupling reaction, benzodioxinoquinoxaline, acridine, phenoxazine, solid-state-enhanced thermally activated delayed fluorescence, room-temperature phosphorescence, organic light-emitting diode

## Abstract

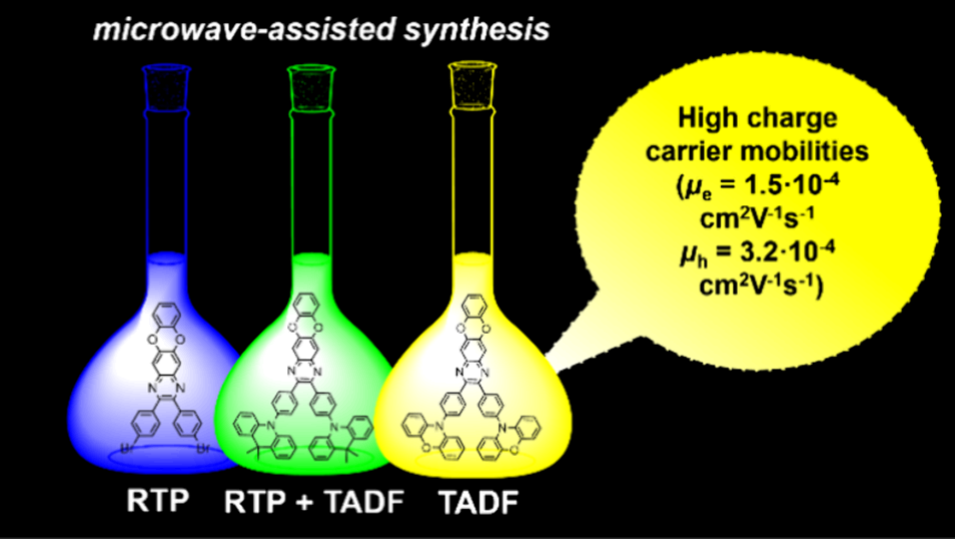

To enhance the usually low-charge carrier mobilities of highly twisted donor–acceptor-type compounds that exhibit thermally activated delayed fluorescence, we designed a rodlike acceptor benzodioxinoquinoxaline. This acceptor and two donor–acceptor–donor derivatives were synthesized via microwave Buchwald–Hartwig cross-coupling reactions with yields of up to 91%. The compounds exhibit three different types of photoluminescence, which is well-explained by quantum chemical calculations. Benzodioxinoquinoxaline shows blue fluorescence, with a very short lifetime of 0.64 ns. Its derivatives exhibit either green solid-state-enhanced thermally activated delayed fluorescence (SSE-TADF) or room-temperature phosphorescence (RTP) with lifetimes approaching 7 ms. When molecularly dispersed in a polymeric host, the compounds show a photoluminescence quantum yield close to 60%. The derivatives containing acridine or phenoxazine moieties exhibit bipolar charge transport. At an electric field of 5.8 × 10^5^ V/cm, hole and electron mobilities of the phenoxazine-containing compound reach 3.2 × 10^–4^ and 1.5 × 10^–4^ cm^2^ V^–1^ s^–1^, respectively. Among the studied SSE-TADF-based organic light-emitting diodes, the device containing this compound shows the highest external quantum efficiency of 12.3% due to the good charge-transporting and SSE-TADF parameters of the emitter.

## Introduction

**Figure 1:**
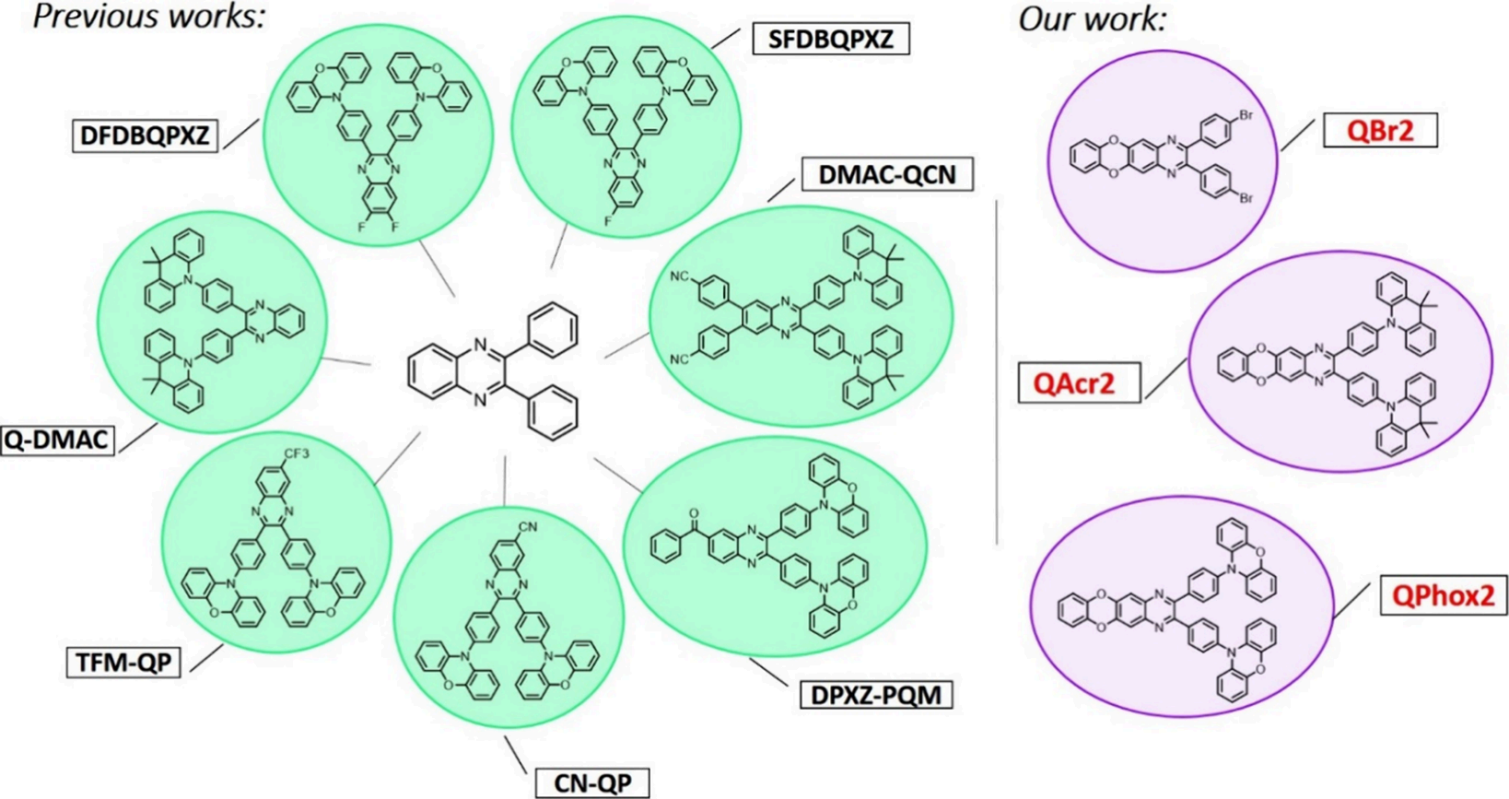
Chemical structures of previously reported and newly developed quinoxaline-containing luminophores.

Quinoxaline-derived compounds are among the most used emitters in the fabrication of efficient yellow, orange, and red OLEDs since they are characterized by inherently narrow band gaps as well as by high thermal and electrochemical stability.^1,2^ The quinoxaline ring can be easily formed via catalyst-free condensation between α-diketones and *ortho*-phenylenediamines, which predetermines wide structural tunability.^3−5^ In the context of structural design, the D–A–D architecture, where electron donors are situated in the *ortho*-position to a quinoxaline acceptor, is beneficial as it imposes a highly twisted molecular configuration that restricts rotational freedom of donor fragments and results in high photoluminescence quantum yields (PLQY) of the compounds.^6^ Phenyl linkers are often incorporated to spatially separate acceptor and donor parts.^7^ The usage of the quinoxaline moiety in the design of organic emitters enables not only to enhance PLQY but also to weaken π–π interactions and initiate solid-state emission enhancement (SSE). The attachment of fluorine atoms to a quinoxaline fragment can enhance the upconversion rate constant (*k*_RISC_) that is advantageous for TADF. The impact of fluorination on SSE-TADF properties of the *N*-phenylphenoxazine-quinoxaline conjugate was studied.^8^ A host-containing OLED with monofluorinated compound SFDBQPXZ (Figure 1) exhibited an EQE of 23.5%, while the device with bifluorinated luminophore DFDBQPXZ (Figure 1) afforded an EQE of 16.8%. The monofluorinated compound was characterized by higher PLQY and lower singlet–triplet energy splitting. The devices based on the emissive layers of the neat compounds demonstrated significantly lower EQEs of 10.1 and 9.8% correspondingly. A host-free OLED based on a nonfluorinated compound that was taken as a reference exhibited an EQE of 8.8%.^8^ Single trifluoromethyl and cyano groups were integrated with the quinoxaline-phenoxazine conjugate.^9^ With the use of these compounds as emitters, not only high EQEs of 14.1% (TFM-QP, Figure 1) and 9.1% (CN-QP, Figure 1) of host-free yellow OLEDs were achieved, but also, single-emissive-layer full-TADF WOLEDs were fabricated. The device containing CN-QP as a yellow emitting compound and DMAC-DPS as a blue emitting TADF compound exhibited an EQE of 20.16%. To strengthen acceptor capacity, another strong electron-accepting moiety, i.e., a benzoyl fragment, was attached to the quinolinyl site of the phenoxazine-carbazole conjugate.^10^ The record EQE value of 26% was achieved for the host-containing device with an electroluminescence intensity maximum at 590 nm and a low turn-on voltage of 2.8 V. Single-emitting-layer WOLEDs based on the quinoxaline-phenanthroline conjugate exhibited an impressive EQE of 32.8%.^11^ When the quinolinyl-accepting group was linked to an acridine donor fragment, the use of the resulting compound (Q-DMAC, Figure 1) as a guest emitter in an OLED afforded an EQE of 12.9% with an electroluminescence (EL) maximum at 512 nm.^12^ The incorporation of two benzonitrile units to the aforementioned D–A conjugate (DMAC-QCN) improved TADF efficiency and allowed the shift of the EL peak of the fabricated host-containing device to the longer wavelength region (545 nm) and to attain an EQE of 17.3%.^13^ Very recently, it was demonstrated that the introduction of a cyclohexyl ring into a quinoxaline-based acceptor resulted in the considerable shift of the PL intensity maxima to the blue spectral region through weakening of the π-conjugation degree.^14^ Such a modification significantly increased the solution processability of the resulting D–A–D compound while maintaining the TADF properties. However, such compounds suffered from a low glass transition temperature. In addition, charge carrier mobilities of highly twisted donor–acceptor-type compounds are usually low.^15,16^

Up to date, most of the reported synthetic procedures for quinoxaline ring formation and quinoxaline functionalization are based on the conventional heating methods, which afford good yields but require a long reaction time.^17,18^ Microwave irradiation is an alternative heating source offering many advantages that cannot be achieved with the aid of conventional heating methods.^19,20^ Microwave irradiation heats the reactor immediately and uniformly, which accelerates reactions by enhancing the collision frequency between molecules. As a consequence, the reaction time is significantly shortened, the unwanted side reactions are minimized, and the target product is obtained in higher yields followed by the simplified purification procedures.

Although there have been known microwave irradiation-assisted (MIA) procedures of quinoxaline synthesis^21,22^ and nucleophilic aromatic substitution of chlorinated quinoxalines,^23^ utilization of quinoxaline-containing intermediates in microwave-assisted Buchwald–Hartwig cross-coupling has not been reported yet.

Herein, we report on the synthesis and properties of three newly designed compounds, 3-bis(4-bromophenyl)benzo[5,6][1,4]dioxino[2,3-*g*]quinoxaline (**QBr2**), 2,3-bis(4-(9,9-dimethylacridin-10(9*H*)-yl)phenyl)benzo[5,6][1,4]dioxino[2,3-*g*]quinoxaline (**QAcr2**), and 2,3-bis(4-(10*H*-phenoxazin-10-yl)phenyl)benzo[5,6][1,4]dioxino[2,3-*g*]quinoxaline (**QPhox2**). They were synthesized in high yields by employing MIA nucleophilic aromatic substitution and MIA Buchwald–Hartwig cross-coupling reactions. All the compounds exhibited excellent thermal stability with the temperatures of the onset of thermal degradation ranging from 330 to 460 °C. Phenoxazine-containing compound **QPhox2** demonstrated bipolar charge-transporting properties with hole and electron mobility values reaching 3.2 × 10^–4^ and 1.5 × 10^–4^ cm^2^ V^–1^ s^–1^, respectively. When being dispersed in a Zeonex matrix and a 3,3′-di(9*H*-carbazol-9-yl)-1,1′-biphenyl (*m*CPB) host, the compound exhibited different channels of the deactivation of triplet excitons, which was theoretically proven by quantum chemical calculations. Bromine-containing compound **QBr2** was found to exhibit room-temperature phosphorescence (RTP), acridine-containing **QAcr2** demonstrated both RTP and TADF, and for **QPhox2**, only TADF was observed. Due to the good charge-transporting ability, the electroluminescence properties of compounds with a D–A–D structure were tested in an OLED utilizing the layers of the molecular mixtures of **QAcr2**/**QPhox2** and *m*CBP as emissive layers. Higher device efficiencies were obtained for an OLED containing phenoxazine derivative **QPhox2** due to the balanced charge carrier mobilities and the TADF-only origin of emission.

## Experimental Section

Computational details and theoretical background, details of synthesis and identifications of compounds, and the corresponding ^1^H NMR spectra are described in detail in the Supporting Information.

### Instrumentation

^1^H and ^13^C NMR spectra were recorded on Varian Unity Plus 400 (400 and 101 MHz, respectively) and Bruker Avance 500 (500 and 126 MHz, respectively) spectrometers in CDCl_3_ solutions, using TMS or the residual peaks of the solvent (2.50 ppm for ^1^H nuclei and 39.5 ppm for ^13^C nuclei) as internal references. Mass spectral analyses were performed using an Agilent 1100 series LC/MSD with the API-ES/APCI mode (200 eV). Elemental analyses were accomplished using a Carlo Erba 1106 instrument. IR spectra were recorded on a Bruker VERTEX 70 FT-IR spectrometer. X-ray single-crystal diffraction was performed on a diffractometer with a CCD detector using a Cu Kα radiation (λ = 1.5418 Å) source.

Using a TA Instruments Q2000, differential scanning calorimetry (DSC) measurements were performed. The samples were examined under a nitrogen atmosphere, and the heating rate was 10 °C/min. Thermogravimetric analysis (TGA) was performed on a TA Instrument Q50. The heating rate was 20 °C/min under a nitrogen atmosphere.

Cyclic voltammetry measurements were performed by using a platinum working electrode (a disk with a diameter of 2 mm) in a three-electrode cell of an Autolab-type potentiostat–galvanostat. The measurements were carried out for the solutions in dry dichloromethane containing 0.1 M tetrabutylammonium hexafluorophosphate at 25 °C; the scan rate was 50 mV/s, while the sample concentration was 10^–3^ M. The potentials were measured against silver as a quasi-reference electrode. A platinum wire was used as a counter electrode. The potentials were calibrated with the standard ferrocene-ferrocenium (Fc/Fc^+^) redox system.

Thin solid films for measurement of UV/vis and PL spectra were prepared by drop casting 2 mg/mL toluene solutions of the compounds on the precleaned quartz substrates. The UV/vis spectra of solutions and thin films of compounds were recorded by a PerkinElmer UV/vis spectrometer Lambda 25. An Edinburgh Instruments FLS980 spectrophotometer and a PicoQuant LDH-D-C-375 laser were used to record the photoluminescence spectra of solutions and thin films and corresponding photoluminescence decays. Phosphorescence spectra of the THF solutions were recorded at 77 K with a delay time of 100 ms.

Vacuum-deposited layers on fluorine–tin oxide-coated glass plates were prepared for recording photoelectron emission spectra for studied compounds performing an experimental setup based on the deep-UV deuterium light source ASBN-D130-CM, a CM110 1/8m monochromator, and an electrometer 6517B Keithley. Organic layers were vacuum-deposited under vacuum of around 2 × 10^–6^ mbar using vacuum equipment from Kurt J. Lesker built in an MB EcoVap4G glovebox. The ionization potentials for the compounds in the solid state were taken from the photoelectron emission spectra. The hole and electron mobilities (μ) for the vacuum-deposited layers were investigated by the time-of-flight (TOF) method using a pulsed Nd:YAG laser (EKSPLA NL300, a wavelength of 355 nm, a pulse duration of 3–6 ns), a Keithley 6517B electrometer, and a Tektronix TDS 3052C. The hole mobility was calculated by utilizing the formula μ = *d*^2^/*U*·*t*_t_, where the transit time (*t*_t_) under applied positive or negative bias (*U*) indicated passage of holes and electrons through the entire thickness (*d*) of the samples.

For the electroluminescence investigations, the prepatterned indium–tin oxide (ITO)-coated glass substrates with seven pixels of a 6 mm^2^ presubstrate were cleaned in an ultrasonic bath. The cleaning process involved successive immersions in deionized water and 2-propanone for 10 min each followed by warming in methanol for 10 min. The current density, voltage, and brightness characteristics were measured using a source meter HP4145A. The measurement of brightness was obtained using a calibrated photodiode, and the Ocean Optics USB2000 spectrometer was used to record electroluminescence spectra.

## Results and Discussion

### Synthesis and X-ray Analysis

**Scheme 1:**
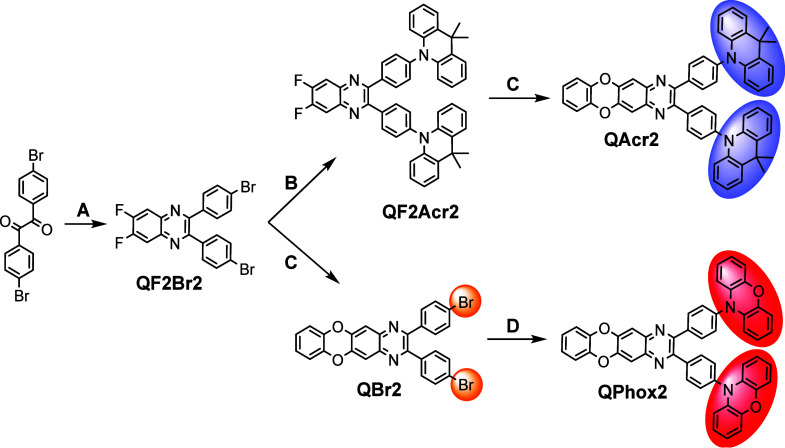
Synthetic Pathways for Compounds **QBr2**, **QAcr2**, and **QPhox2** Conditions: (A) 4,5-difluorobenzene-1,2-diamine, CH_3_COOH, reflux for 24 h; (B) 9,9′-dimethyl-9,10-dihydroacridine, NaO^t^Bu, XPhos, Pd_2_(dba)_3_, dry toluene, microwave irradiation for 30 min, 130°C; (C) pyrocatechol, K_2_CO_3_, dry DMF, microwave irradiation for 30 min, 130°C; (D) phenoxazine, NaO^t^Bu, XPhos, Pd_2_(dba)_3_, dry toluene, microwave irradiation for 30 min, 130°C.

The chemical structures of the designed compounds as well as the synthetic pathways are depicted in Scheme 1. The formation of tetrahalogenated intermediate **QF2Br2** was achieved via calalyst-free condensation between 4,4-dibromobenzil and 4,5-difluorobenzene-1,2-diamine. Generally, condensation of 1,2-diketones and 1,2-diamines occurs easily; however, functionalization of 1,2-diamines with electron-withdrawing substituents leads to the slightly lower yields and the prolonged reaction time.^24^ For this reason, the formation of a quinoxaline cycle was carried out in refluxing glacial acetic acid for 24 h and afforded the key intermediate **QF2Br2** in the yield of 92%. Compound **QBr2** was synthesized via C–F bond cleavage/C–O bond formation of **QF2Br2** with pyrocatechol. The reaction was carried out under microwave irradiation at 100 °C for 30 min in dry DMF and with an excess amount of potassium carbonate. The target compound **QPhox2** was synthesized via a microwave irradiation-assisted Buchwald–Hartwig cross-coupling reaction between **QBr2** and 10*H*-phenoxazine in 73% yield. Acridine-containing compound **QAcr2** was obtained through the opposite synthetic route. First, a microwave irradiation-assisted Buchwald–Hartwig cross-coupling reaction was carried out between **QF2Br2** and 9,9-dimethyl-9,10-dihydroacridine to afford compound **QF2Acr2** in 86% yield. The following nucleophilic aromatic substitution of **QF2Acr2** with pyrocatechol under microwave irradiation yielded compound **QAcr2** in 91% yield. The target compounds demonstrated good solubility in common organic solvents, such as toluene, chloroform, acetone, and THF. The structures of the synthesized compounds were confirmed by ^1^H and ^13^C NMR spectroscopies and mass spectrometry (Supporting Information).

**Figure 2:**
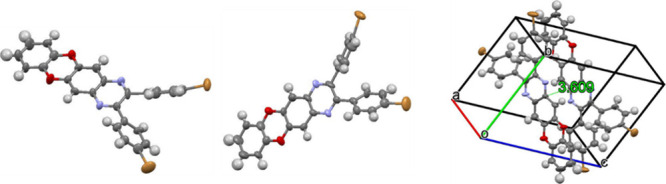
(a) X-ray crystal structure and geometry of compound **QBr2** from different perspectives and (b) packing pattern of compound **QBr2** in the solid state.

The single crystal of compound **QBr2** suitable for X-ray analysis was grown by slow evaporation from a mixture of hexane and ethyl acetate, whereas the sizes of crystals of compounds **QAcr2** and **QPhox2** were too small to perform X-ray analysis. Figure 2a represents the geometry of a single molecule form different perspectives, while Figure 2b demonstrates the packing of **QBr2** in a single crystal. The analysis of the crystallographic data shows that compound **QBr2** crystallizes in a triclinic crystal system that belongs to space group *P1̅*. Compound **QBr2** was capable of participation in various types of intermolecular interactions that occurred mainly between molecules of bromine-substituted phenyl rings and condensed benzodioxinoquinoxaline moieties. These are the following: C–H···Br interactions with the distance of 3.000 Å, C···Br interactions with the distance of 3.531 Å, C–H···C interactions between bromine-substituted phenyl rings and oxygen-containing rings with the distance of 2.738 Å, Br···Br interactions between neighboring bromine-substituted phenyl rings with the distance of 3.367 Å, C–H···O interactions with the distance of 2.541 nm, and C–H···N interactions with the distance of 2.665 Å. In addition, the neighboring benzodioxinoquinoxaline fragments are located parallel to each other with the distance of 3.609 Å, which is evidence of π–π stacking. Such an arrangement of the molecules of **QBr2** in the solid state has an impact on its luminescence quantum yield as is discussed below.

### Computational Studies

**Figure 3:**
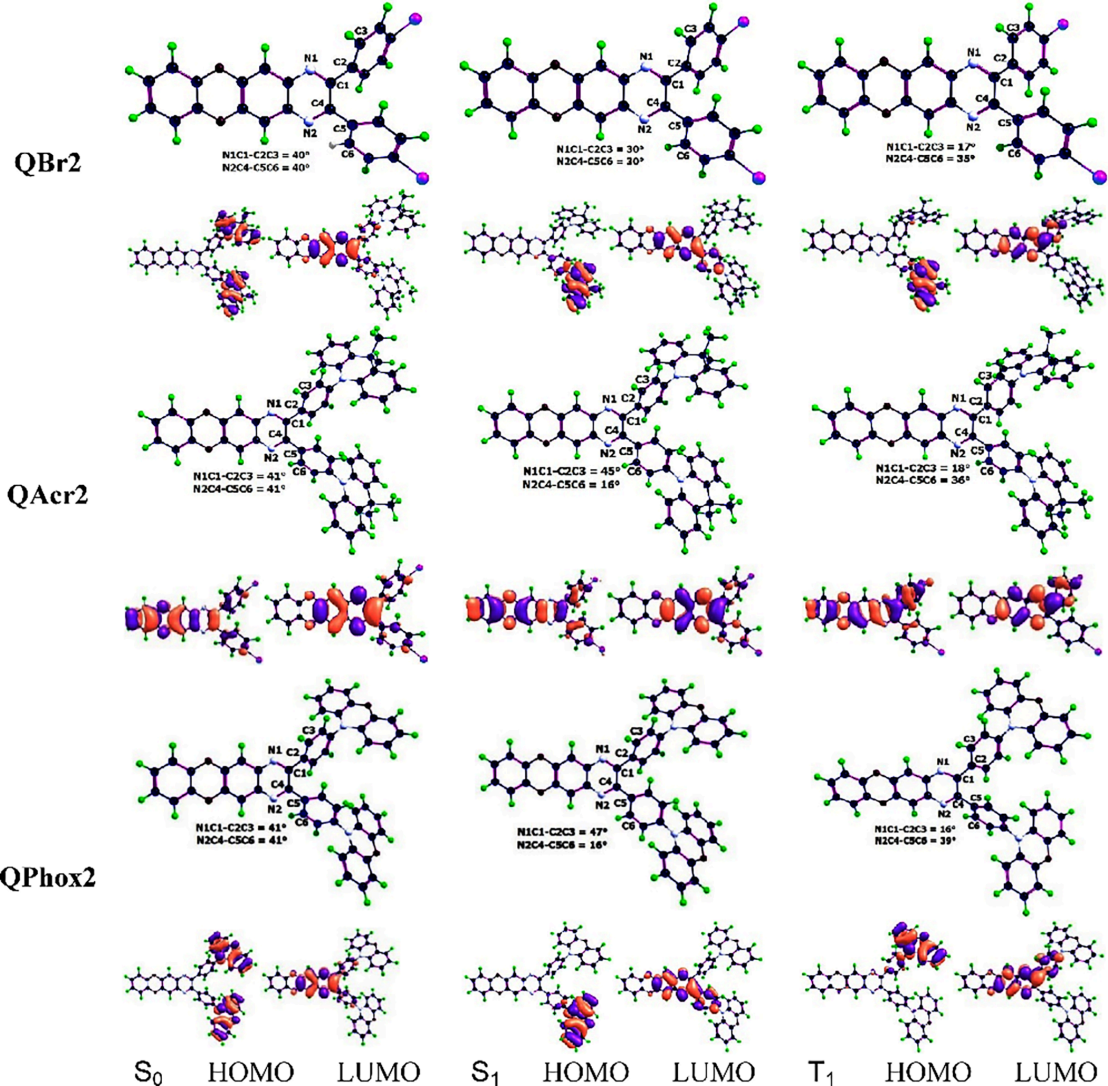
S_0_, S_1_, and T_1_ state optimized geometries and frontier molecular orbitals of **QBr2**, **QAcr2**, and **QPhox2** in toluene.

The molecular structures were optimized in the ground state (S_0_) at the density functional theory (DFT) level for the toluene and benzene solutions using the B3LYP functional and the 6-31G(d) basis set. The first excited singlet (S1) and triplet (T1) states were optimized using CAM-B3LYP with the 6-31G(d) basis set employing TD-DFT with the Tamm–Dancoff approximation (TDA). The LC-ωPBE functional with the 6-31G(d) basis set within the TDA formalism was used to compute the wavelengths of fluorescence (λ_flu_) and phosphorescence (λ_phos_). The detailed computational methodology is given in the SI. Figure 3 shows the S_0_, S_1_, and T_1_ state geometries of **QBr2**, **QAcr2**, and **QPhox2** molecules optimized in toluene solution (more details in the SI). The torsion angle between the D and A units of the molecule is one of the key factors determining the performance of TADF-based OLEDs, which affects the oscillator strength and spin–orbit coupling as well as the singlet–triplet energy gap.^25−27^ As a consequence, the torsion angle significantly influences the intersystem crossing (ISC) and reverse intersystem crossing (RISC) rate constants.^28,29^ In terms of torsion angles between A and two same space units, i.e., *D*(N1C1–C2C3) and *D*(N2C4–C5C6) (Figure 3), the optimized S_1_ and T_1_ state geometries differ from those of the ground singlet state for the three molecules with substantial deviations for the toluene solutions (Figure 3). Both D–A dihedrals are the same for the S_0_ state geometries due to the symmetrical structure of the molecules of **QBr2**, **QAcr2**, and **QPhox2**. However, the symmetry is broken for S_1_ and T_1_ state geometries due to the localization of the corresponding excited state on the particular D–A branch (the only exception is the S_1_ state of **QBr2**, which sustains a symmetric structure similar to the S_0_ state). These structural variations strongly affect the computed energetic characteristics, such as reorganization energies and Δ*E*_ST_. The significant values of the oscillator strength (*f,* see Table 1) of the S_0_←S_1_ transition show the strong fluorescence nature of all the molecules at the S_1_ optimized molecular geometry (Table 1).

**Table 1:** Spectroscopic Characteristics of Compounds **QPhox2**, **QAcr2**, and **QBr2** Calculated by the LC-ωPBE*/6-31G(d) Method with Optimally Tuned Range-Separation Parameter (ω) Values and the PCM Solvent Model in Comparison with Experimental Data^a^

molecule	ω, Bohr^–1^	λ_exp_, nm, SL + Zeonex film (vacuum)	λ_exp_, nm	medium	λ_flu_, nm (*f*)	λ_phos_, nm	Δ*E*_ST_, eV
**QPhox2**	0.175	485	570	toluene	483 (0.189)	635	0.38
			580	benzene	484 (0.173)	635	0.20
**QBr2**	0.185	436	424	toluene	388 (0.798)	624	0.57
			425	benzene	388 (0.799)	507	0.67
**QAcr2**	0.17	460	510	toluene	438 (0.579)	626	0.62
			515	benzene	439 (0.567)	626	0.62

a The oscillator strength (*f*) values are provided in parentheses alongside the corresponding wavelengths of fluorescence intensity maxima.

The results of the analysis of the frontier molecular orbitals allow to explain the significant values of the oscillator strength for the S_0_←S_1_ transition in terms of nonzero expansion coefficients on the common benzene linker between D and A (Figure 3).

We performed TDA-DFT calculations to compute the luminescence properties of the three molecules. Table 1 lists the wavelengths of fluorescence (λ_flu_), phosphorescence (λ_phos_), and adiabatic singlet–triplet energy gaps (Δ*E*_ST_) computed with the LC-ω*PBE functional. The computed λ_flu_ values for toluene and benzene solutions are approximately in line with the experimental results (λ_exp_). The very large Δ*E*_ST_ gaps estimated for compounds **QBr2** and **QAcr2** (Table 2) allow us to assume that these compounds should not show TADF, while **QPhox2** sustains a smaller Δ*E*_ST_ gap suitable for the TADF effect.

**Table 2:** Theoretical Photophysical Characteristics of Compounds **QPhox2**, **QAcr2**, and **QBr2** in Benzene Solutions

molecule	⟨S_1_|Ĥ_SO_|T_1_⟩, cm^–1^ (at S_1_ geom)	⟨S_1_|Ĥ_SO_|T_1_⟩, cm^–1^ (at T_1_ geom)	λ_S_, eV	λ_T_, eV	*k*_flu_, s^–1^	*k*_phos_, s^–1^	*k*_ISC_, s^–1^	*k*_RISC_, s^–1^
**QPhox2**	0.21	0.56	0.227	0.197	1.85 × 10^7^	1.87	2.56 × 10^7^	2.28 × 10^4^
**QBr2**	0.63	0.73	0.022	0.006	3.32 × 10^8^	10.65	≈0	4.70 × 10^–88^
**QAcr2**	0.51	0.84	0.231	0.104	7.45 × 10^7^	0.402	9.68 × 10^1^	7.02 × 10^–6^

We estimated the rates of ISC (*k*_ISC_) and RISC (*k*_RISC_) for benzene solutions within the semiclassical Marcus theory expression using the computed spin–orbit coupling matrix elements (SOCMEs), λ_S_ and λ_T_ energy values, and Δ*E*_ST_ gaps (Table 2). For **QPhox2**, the fluorescence (*k*_flu_) and phosphorescence (*k*_phos_) rate constants are predicted as 1.85 × 10^7^ and 1.87 s^–1^, respectively. The corresponding values for **QAcr2** are 7.45 × 10^7^ and 0.402 s^–1^, respectively. The tiny *k*_phos_ values show a weak and long-lived slow phosphorescence for all three compounds in case that nonradiative quenching of the T_1_ state is suppressed. The predicted rates of ISC (*k*_ISC_) and RISC (*k*_RISC_) for **QPhox2** are 2.56 × 10^7^ and 2.28 × 10^4^, respectively (Table 2). This means that the estimated rate of ISC is comparable to that of the fluorescence rate constant *k*_flu_ for **QPhox2**, and hence, ISC is competitive to the prompt fluorescence process. The rate-determining step of TADF is RISC. The ratio of *k*_ISC_ to *k*_RISC_ essentially describes the TADF efficiency for **QPhox2** and demonstrates slow TADF (τ_exp_ = 2.8 ms). The phosphorescence rate of **QPhox2** is negligible and cannot compete with the fluorescence channel under room-temperature conditions. Based on the incredibly small computed values of *k*_RISC_ for **QAcr2** (7.02 × 10^–6^ S^–1^) and **QBr2** (4.70 × 10^–88^ S^–1^), Table 2 indicates that they do not show TADF. The small value of *k*_ISC_ estimated for **QAcr2** shows the slow intersystem crossing phenomenon and possibly causes the efficient prompt fluorescence (*k*_flu_ = 7.45 × 10^7^ s^–1^, see Table 2). This observation is in agreement with the experimental observations. The long-lived phosphorescence was observed for compounds **QBr2** and **QAcr2**, while for **QAcr2**, the redshifted and extremely slow TADF (τ_exp_ = 56 ms) was also found, probably due to the structural distortions of some molecules with respect to larger *D*(N1C1–C2C3) and *D*(N2C4–C5C6) dihedrals in the Zeonex matrix.

### Charge-Transporting, Thermal, Electrochemical, and Photoelectrical Properties

#### Charge-Transporting Properties

**Figure 4:**
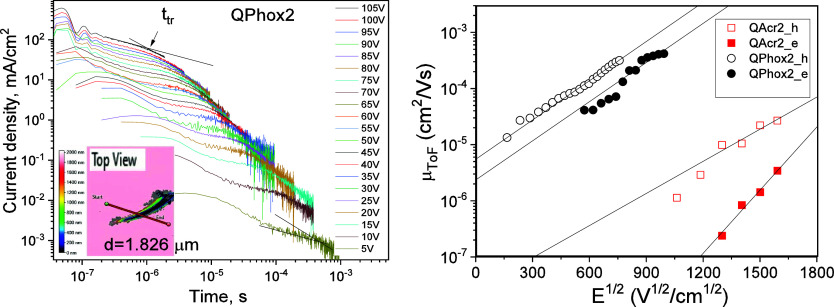
(a) TOF signals recorded for the thick film of **QPhox2** at the different voltages (the inset shows data from the thickness measurement (Figure S2)). (b) Hole (h) and electron (e) mobility values of **QAcr2** and **QPhox2** versus the electric field (the lines were obtained by the Poole–Frenkel electric field dependence).

The layers of **QAcr2** and **QPhox2** for the TOF measurements were deposited^30^ (Figure S1). The transport of holes and electrons was detected for **QAcr2** and **QPhox2**, by applying positive or negative voltages (V), respectively, to the optically transparent electrode. The shortened current transient curves were recorded at increasing electric fields due to the fast drifts of photogenerated charge carriers across the layers. Due to the low dispersity of hole transport of **QPhox2** (Figure 4a), the current transients with easily detectable transit times (*t*_tr_) were recorded starting from low electric fields of 2.7 × 10^4^ V/cm. Because of the high dispersity of charge carrier transport of **QAcr2**, it was difficult to observe transit times at electric fields lower than 1 × 10^6^ V/cm. The hole and electron mobility values μ_h,e_ for the films of **QAcr2** and **QPhox2** were calculated by using the formula μ_h,e_ = *d*^2^/(*V·t*_tr_). The thicknesses (*d*) of the tested TOF samples of **QAcr2** and **QPhox2** were measured by the profilometer Profilm3D (Figure S2). The good-quality TOF samples were not obtained for **QBr2** due to the crystallinity of its layers. Hole and electron mobilities reached 3.2 × 10^–4^ and 1.5 × 10^–4^ cm^2^ V^–1^ s^–1^, respectively, at the electric field of 5.8 × 10^5^ V/cm for the film of phenoxazine-containing compound **QPhox2**. At a considerably higher electric field of 2.5 × 10^6^ V/cm, hole and electron mobilities reached only 2.7 × 10^–5^ and 3.5 × 10^–6^ cm^2^ V^–1^ s^–1^, respectively, for the acridine derivative **QAcr2**. The zero mobilities (μ_0e,h_) and field-dependent parameters (β_e,h_) for **QAcr2** and **QPhox2** were found to be different according to fitting by the Poole–Frenkel electric field dependence μ_e,h_ = μ_0e,h_·exp(β_e,h_·*E*^1/2^) (Table 3). This result can be attributed to the different donor substituents of benzodioxinoquinoxaline. When **QAcr2** and **QPhox2** were used for the fabrication of the OLEDs, the same trend was observed in device efficiencies despite close values of PLQYs of neat films of the compounds (Table 3). The considerably higher maximum EQE of 12.3% of **QPhox2**-based OLEDs in comparison to that of 1.63% of **QAcr2**-based OLEDs was obtained mainly because of the very different charge-transporting properties of **QAcr2** and **QPhox2** (see the last section).

**Table 3:** Charge-Transporting, Thermal, Electrochemical, and Photoelectrical Parameters of **QBr2**, **QAcr2**, and **QPhox2**

	**QBr2**	**QAcr2**	**QPhox2**
μ_0h_, cm^2^ V^–1^ s^–1^		6.5 × 10^–12^	5.5 × 10^–6^
μ_0e_*,* cm^2^ V^–1^ s^–1^		2.3 × 10^–14^	2.4 × 10^–6^
β_h_, V^0.5^ cm^–0.5^		4.4 × 10^–3^	5.4 × 10^–3^
β_e_, V^0.5^ cm^–0.5^		8.6 × 10^–3^	5.3 × 10^–3^
*T*_d_^5%^, °C^a^	330	432	460
*T*_g_, °C^b^		158^g^	
*T*_m_, °C^c^	306^e^^,^^g^	263^e^, 286^e^^,^^g^	335^e^
*T*_cr_, °C^d^	293^f^	219^g^	299^f^
*E*_ox_^onset^, V	1.16	0.48	0.3
IP^CV^, eV	6.26	5.58	5.4
*E*_red_^onset^, V	2.08	2.08	2.08
EA^CV^, eV	3.02	3.02	3.02
*E*_G_^CV^, eV	3.24	2.56	2.38
IP^PE^, eV		5.66	5.3
*E*_g_, eV	2.88	2.76	2.69
EA^PE^, eV		2.9	5.61

a Temperature of 5% weight loss, determined from TGA curves.

b Glass transition temperature.

c Melting point.

d The temperature of crystallization.

e Determined from the first heating scan.

g Determined from cooling scan.

f Determined from the second heating scan.

#### Thermal Transitions

High thermal stability of the compounds and morphological stability of their films are indispensable points for electroactive compounds to be used in OLEDs, as a guarantee of the stability of their thin layers. In order to evaluate thermal stability as well as morphological transformations of the newly synthesized quinoxaline-based compounds, thermogravimetric analysis (TGA) and differential scanning calorimetry (DSC) measurements were performed (Figure S3). The corresponding data are summarized in Table 3. All of the compounds demonstrated excellent thermal stability with the 5% weight loss temperature (*T*_d_^5%^) above 330 °C (Figure S3a). Phenoxazine-containing compound **QPhox2** demonstrated the highest thermal stability with a *T*_d_^5%^ of 460 °C. Acridine-containing compound **QAcr2** revealed a *T*_d_^5%^ of 432 °C. The higher thermal stability of the latter compound can be attributed to more intensive intermolecular interactions in the solid state. The complete weight loss of **QBr2** during the TGA experiment indicates that the compound underwent sublimation. The target compounds were obtained as crystalline substances after the synthesis. The DSC experiment proved their crystallinity. During the first DSC scan, both compounds **QBr2** and **QPhox2** demonstrated single endothermic melting signals at 306 and 335 °C, respectively. The same signals appeared in the repeated heating scans. During the cooling scans, compounds **QBr2** and **QPhox2** revealed crystallization peaks at 293 and 299 °C, correspondingly. No glass transition was observed for either of the compounds (Figure S3b). During the first heating scan, compound **QAcr2** exhibited two endothermic melting signals at 263 and 286 °C that could be ascribed to the formation of different types of polymorphs. Cooling down of the melt revealed glass transition at 158 °C, thus indicating that compound **QAcr2** is capable of molecular glass formation. The second heating of compound **QAcr2** revealed a crystallization peak at 219 °C and melting at 286 °C (Figure S3b).

#### Electrochemical Properties

**Figure 5:**
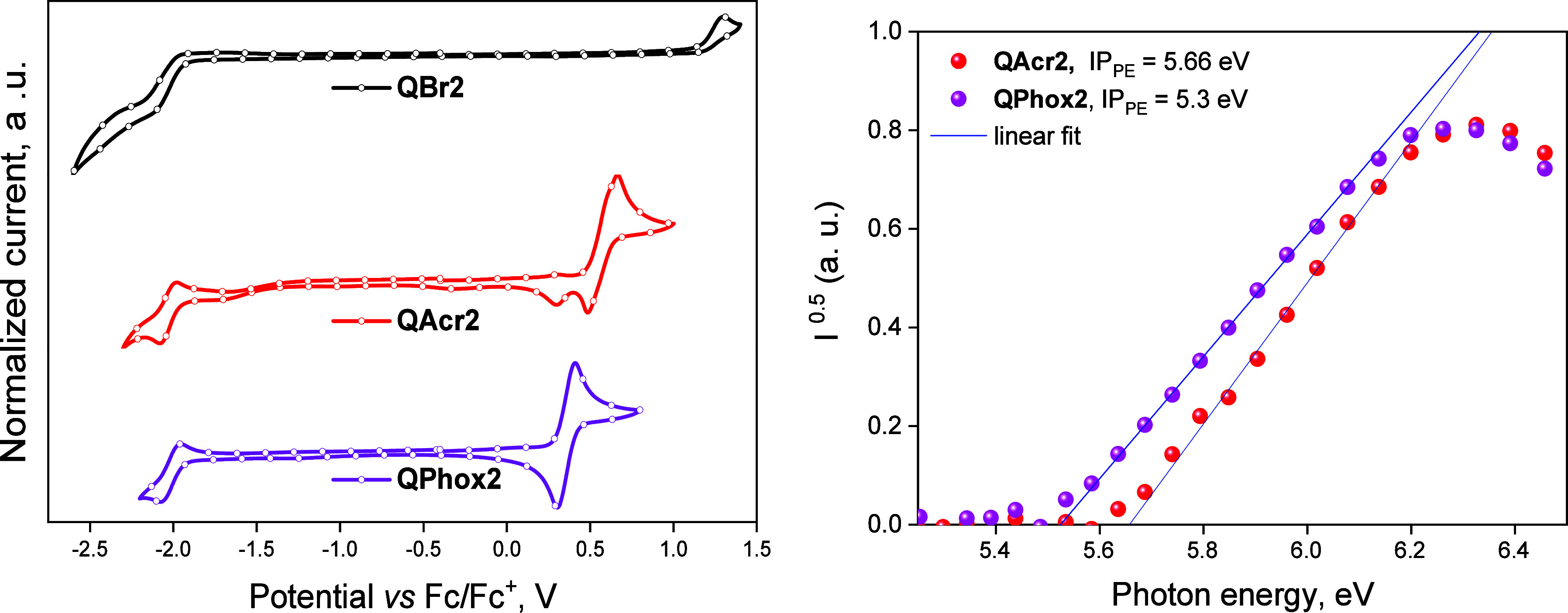
(a) Cyclic voltammograms of compounds. (b) Photoelectron emission spectra of vacuum-deposited films of compounds **QAcr2** and **QPhox2.**

To determine the energy levels of the synthesized compounds, cyclic voltammetry (CV) measurements were carried out. The measurements were performed for dichloromethane solutions containing tetra-*n*-bytulammonium hexafluorophosphate (TBAPF_6_) as a supporting electrolyte. A ferrocene/ferrocenium redox couple was used as an internal standard to calculate oxidation and reduction onsets (*E*_ox_^onset^ and *E*_red_^onset^). The voltammograms are depicted in Figure 5a. The corresponding data are summarized in Table 3. During anodic scan, compound **QBr2** exhibited a single irreversible oxidation wave with an *E*_ox_^onset^ value of 1.16 V. Compound **QAcr2** revealed quasi-reversible oxidation (*E*_ox_^onset^ = 0.48 V) that is common for acridine-containing compounds with unsubstituted C-3 and C-6 positions of acridine.^31^ Compound **QPhox2** exhibited reversible oxidation at 0.3 V. During cathodic scan, all the compounds demonstrated single reversible reduction waves with an *E*_red_^onset^ value of 2.07 V due to the presence of the same benzodioxinoquinoxaline electron-withdrawing moiety. In order to evaluate ionization potentials (IP^CV^) as well as electron affinities (EA^CV^), the equations IP^CV^ = 5.1 eV + *E*_ox_^onset^ and EA^CV^ = EA^CV^ = 5.1 eV – *E*_red_^onset^ were used.^32^ Bromine-containing compound **QBr2** exhibited the highest IP^CV^ of 6.26 eV. Compound **QAcr2** was characterized by an IP^CV^ value of 5.58 eV that is slightly higher than that of **QPhox2** (IP^CV^ = 5.4 eV). This observation can be attributed to the stronger electron-donating ability of phenoxazine. For all the compounds, the EA^CV^ values were found to be 3.02 eV.

As long as the target compounds were designed to be utilized in solid-state optoelectronic devices, it was important to evaluate their solid-state ionization potentials (IP^PE^). The IP^PE^ values of the solid films of the investigated compounds were obtained by photoelectron emission (PE) spectroscopy. In accordance with the following relation *i* ≈ (*h*ν – IP^PE^)^2^, the current (*i*) versus photon energy (*h*ν) was plotted when *I* was represented in the quadratic model and *h*ν in the linear model (Figure 5b). The linear fitting of the plotted data revealed IP_PE_ values of compounds **QAcr2** and **QPhox2** to be of 5.69 and 5.52 eV correspondingly (Table 3). These values were inconsiderably higher than those taken from the CV measurements. The trend of the decrease in IP^PE^ with the increasing donor strength was maintained.

### Photophysical Properties

**Figure 6:**
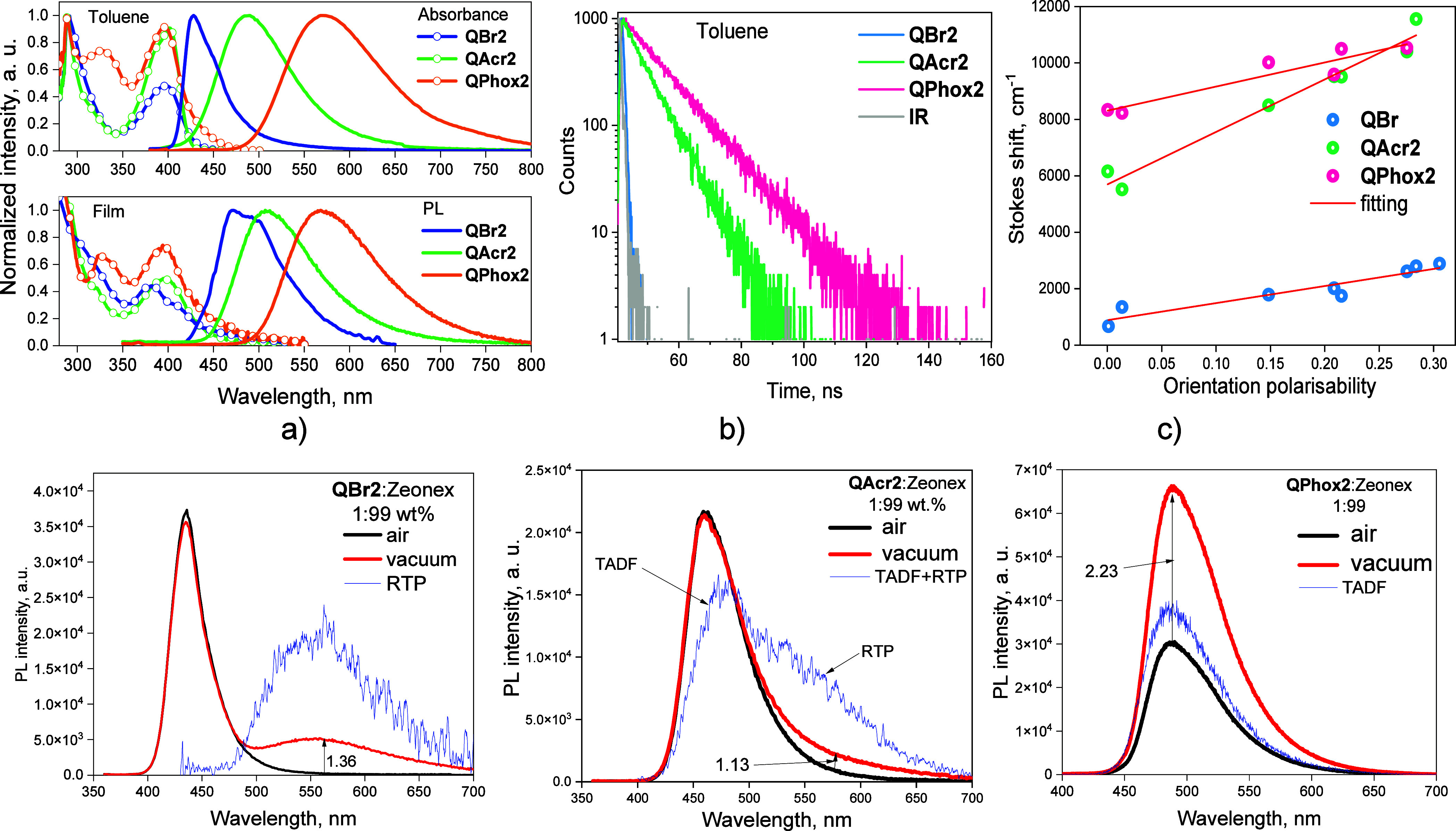
Absorption and PL spectra (a) of toluene solutions and films of compounds **QBr2**, **QAcr2**, and **QPhox2**; PL decay curves (b) of the deoxygenated toluene solutions; Lippert–Mataga plots (c) of the compounds; PL spectra (d–f) of 1 wt % molecular dispersions of **QBr2**, **QAcr2**, and **QPhox2** in Zeonex, recorded in air and vacuum. The thin blue curves are spectra recorded with a delay of 1 ms after excitation.

The investigation of the photophysical properties of compounds **QBr2**, **QAcr2**, and **QPhox2** was started from their dilute solutions and thin vacuum-deposited films (Figure 6a and Figure S4). The acceptor **QBr2** absorbed light up to 450 nm. The absorption spectra of **QBr2** were characterized by two major absorption bands at about 290 and 400 nm. These bands mainly refer to the absorption of locally excited (LE) bands of benzodioxinoquinoxaline. The same bands were recognized for **QAcr2** and **QPhox2**. The phenoxazine-containing **QPhox2** showed the additional well-expressed absorption band at ca. 330 nm (Figure 6a). As it was reported previously for *N*-phenyl-substituted phenoxazine,^33^ such a band originates predominantly from LE states of the phenoxazine moiety with the minor charge transfer (CT) component from phenoxazine to the adjacent phenyl ring. The absorption spectrum of acridine-containing compound **QAcr2** differs from that of **QBr2** by the shoulder at ca. 320 nm (Figure 6a). This shoulder originates from LE absorption of acridine.^34^ The band with a maximum at ca. 290 nm was not sensitive to the polarity of the solvent. It is attributed to π–π* transitions of aromatic rings of benzodioxinoquinoxaline. The low-energy bands (LEBs) demonstrated a slight shift (∼10 nm) with the increasing polarity of the solvents (Figure S4a–c). Such shifts can be explained by overlapping of closely located polarity-independent LE and polarity-dependent CT absorption bands of **QAcr2** and **QPhox2**. The toluene solutions of **QBr2**, **QAcr2**, and **QPhox2** were characterized by the molar absorption coefficients (ε) of 88,041, 84,439, and 71,607 M^–1^cm^–1^, respectively (Table 4 and Table S2). The values of ε decreased to 37,133, 39,289, and 22,609 M^–1^ cm^–1^, respectively, for DMF solutions (Table S2). Interestingly, compound **QBr2**, even though lacking a typical donor–acceptor architecture, demonstrated the LEBs with the same trend as **QAcr2** and **QBPhox2**, both bearing a donor–acceptor structure. The LEBs of the film of **QBr2** also demonstrated the vibronic structure, in contrast to the toluene solution (Figure 6a). Apparently, such a vibronic structure can be attributed to the restriction of movement of the phenyl rings in the solid state. The nonstructured LEBs of the solutions were recorded when the molecular movements were allowed (Figure S4a–c).

**Table 4:** Photophysical Parameters of **QBr2**, **QAcr2**, and **QPhox2**^a^

	**toluene**					**Zeonex (air/vac)**		**Δ*E*_ST_, eV**	
compound	**λ**_**abs**_**, nm**	**ε**, M^–1^ cm^–1^	**λ**_**em**_**, nm**	**Δν, cm**^**–1**^	**PLQY, %**	**PLQY, %**	**τ**_**1**_**/τ**_**2**_**, ns/ms**	THF	mCBP
**QBr2**	392	88,041	428	1980	15	9/12	0.65/6.1^RTP^	0.53	0.36
**QAcr2**	390	84,439	485	6147	17	30/34	5.2/56^TADF^, 7^RTP^	0.34	0.1
**QPhox2**	388	71,607	570	8229	23	27/60	8.6/2.8^TADF^	0.18	0.08

a λ_abs_, wavelengths of absorbance maxima; ε, molar extinction coefficient; Δν, Stokes shift; PLQY, photoluminescence quantum yield; τ_1_, the lifetime of the prompt component of photoluminescence decay; τ_2_, the lifetime of the delayed component of photoluminescence decay; Δ*E*_ST_, singlet–triplet energy splitting.

PL spectra of the toluene solutions of compounds **QBr2**, **QAcr2**, and **QPhox2** were found to be structureless with intensity maxima at 427, 485, and 570 nm correspondingly (Figure 6a and Table 4). The fluorescence lifetimes observed for toluene solutions increased with changing the molecular structure from **QBr2** and **QAcr2** to **QPhox2** (Figure 6b). The similar trend was observed for the oxygen sensitivity of **QBr2**, **QAcr2**, and **QPhox2** (Figure S4). The ratios of 1.1, 2.1, and 2.7 of fluorescence intensities for deoxygenated to oxygenated toluene solutions of **QBr2**, **QAcr2**, and **QPhox2** were obtained, respectively (Figure S5). This observation can be attributed to the different efficiencies of the ISC processes. Molecular oxygen, which is known to appear in the ground triplet state, quenches the emissive triplet states of luminophores through triplet energy interception.^35^ When the fast relaxations of triplets by oxygen were restricted, the fluorescence lifetimes of the toluene solution of **QPhox2** increased from 9.87 to 12.53 ns (Figure S4).

For parent compound **QBr2**, no significant shift in PL maxima was observed, with the change in the polarity of the media indicating that its luminescence originated mainly from LE states. The change in the solvents from low-polarity toluene to highly polar tetrahydrofuran resulted in the bathochromic shift of PL maxima of the solutions of compounds **QAcr2** and **QPhox2** significantly. The shift of 116 nm was observed for the solutions of **QAcr2** and of 112 nm for the solutions of **QPhox2**. This observation indicates that the origin of the emission of **QAcr2** and **QPhox2** is intramolecular charge transfer. Ten solvents of different polarities were selected to investigate the solvatochromic behavior of the compounds. The plot of Stokes shifts (Δν =ν_abs_ – ν_em_) versus orientation polarizability of solvents (Δ*f*) that is described by the Lippert–Mataga equation is presented in Figure 6c. The calculated slopes are associated with the change in the dipole moment of the compound in the ground (μ_g_) and excited (μ_e_) states. They characterize the specific polarity of the luminophore that originates from the unimolecular combination of electron-donating and electron-accepting moieties. For compound **QBr2**, the linear dependence with the slope of 6051 cm^–1^ was obtained. It is typical for compounds with predominant emission from locally excited states. The acridine-containing compound (**QAcr2**) was characterized by the highest slope of Lippert–Mataga dependence in the series of 18,606 cm^–1^. This observation points out to the strongest CT character of emission. The values close to that are common for conventional TADF emitters.^36,37^ The emission of compound **QPhox2** was found to be less sensitive to the polarity of the solvent. It was characterized by a much lower slope of 8307 cm^–1^, presumably due to the simultaneous contribution of LE and CT into the emission process. The tendency of the ascending CT character of emission with the increase in dipole moments is mirrored in PLQY values of the solutions of the compounds in toluene. PLQYs were estimated to be 11.8, 15.3, and 12.6% for the solutions of quinoxaline derivatives **QBr2**, **QAcr2**, and **QPhox2**, respectively (Table S3). PLQYs of neat films of the studied compounds were estimated to be 2.8, 15.7, and 13.1% for **QBr2**, **QAcr2**, and **QPhox2,** respectively. This points out that the PLQY of the target compounds may be influenced by the formation of aggregates, which is discussed in more detail. The emission profiles of neat films of **QAcr2** and **QPhox2** were structureless, with no detectable contribution of LE. The PL intensity maxima were located at 510 nm for **QAcr2** and at 569 nm for **QPhox2** (Figure 6a). The structured PL spectrum with maxima at 470 and 497 nm was recorded for the film of **QBr2**. The PL intensity of the film of the parent compound **QBr2** did not change upon deoxygenation. Meanwhile, the PL intensities of the films of compounds **QAcr2** and **QPhox2** increased slightly after removal of oxygen from the media, suggesting the involvement of triplet excited states in the emission (Figure S6a–c). However, PL decay curves of the films of the compounds were adequately fitted in the nanosecond range by using a single-exponential mathematical model, and no delayed component was observed under oxygen-free conditions (Figure S6d–f). Therefore, it can be concluded that the contribution of triplet excitons in the emission of neat films of the studied compounds is too inconsiderable to suspect TADF.

**Figure 7:**
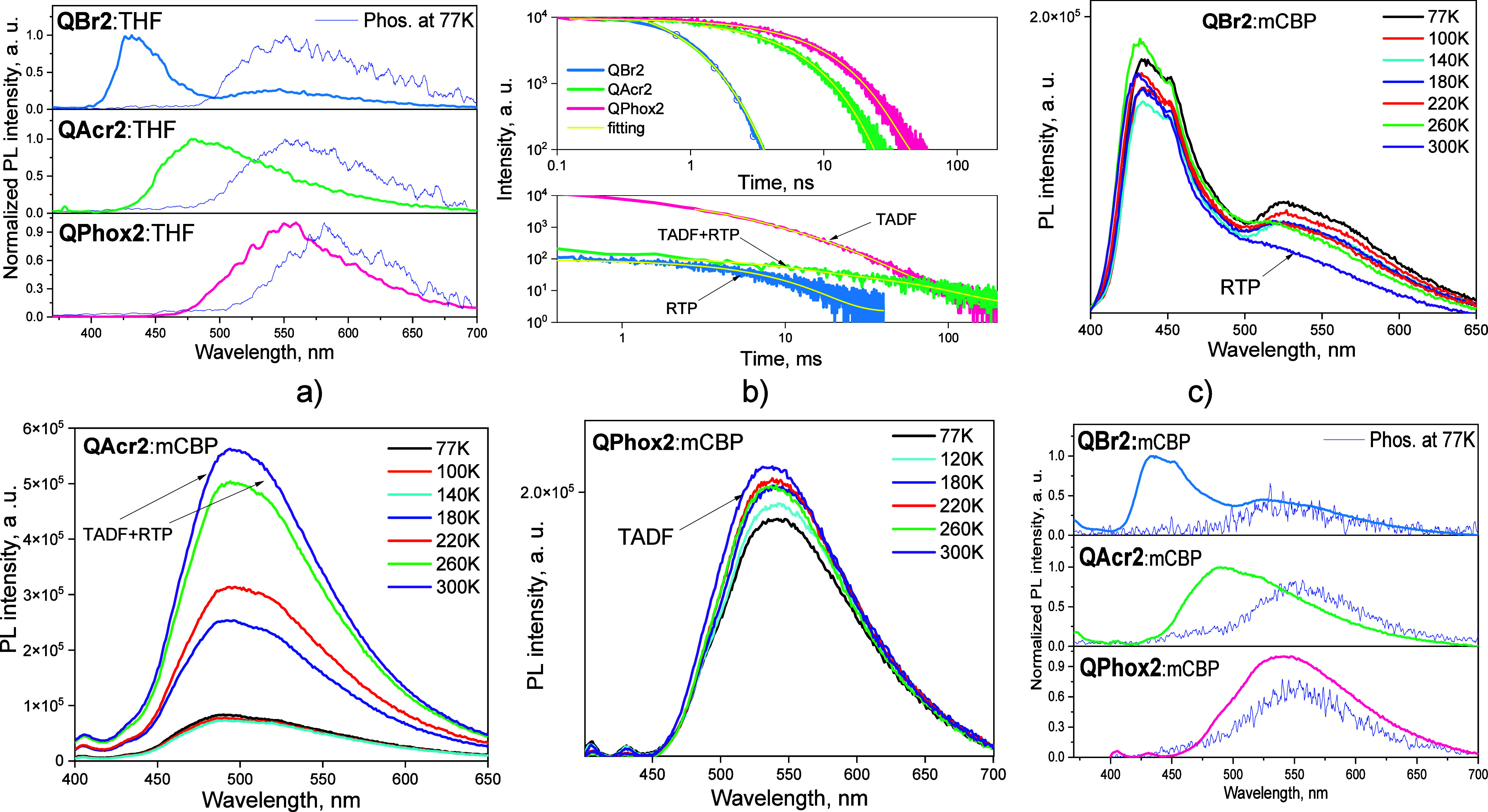
PL and phosphorescence (Phos.) spectra (a) of frozen THF solutions of **QBr2**, **QAcr2**, and **QPhox2** recorded at 77 K. PL decay curves (b) of 1 wt % molecular dispersions of **QBr2**, **QAcr2**, and **QPhox2** in Zeonex collected in nanosecond and millisecond ranges. PL spectra (c–e) of 10 wt % molecular dispersions of compounds **QBr2**, **QAcr2**, and **QPhox2** in *m*CBP recorded at the different temperatures. Phos. spectra were recorded with a 100 ms delay after excitation. PL and phosphorescence (Phos.) spectra (f) of 10 wt % molecular dispersions of the compounds in *m*CBP at 77 K.

To establish whether the luminescence intensities of solutions of the investigated compounds are influenced by the participation of triplet excited states, the PL spectra of air-equilibrated, saturated by oxygen, and deoxygenated toluene solutions of compounds were recorded (Figure S5). As briefly mentioned above, the PL intensities of the solutions of compounds **QAcr2** and **QPhox2**, as well as those of the corresponding PL decays (Figure S5), increased after the removal of oxygen. For the solution of **QPhox2**, the increment was the most noticeable. The PL intensity of a toluene solution of bromine-containing compound **QBr2** showed almost no dependence on the concentration of oxygen. Participation of triplet excited states in the emission could be the evidence of RTP and TADF, which occur via ISC or RISC processes, respectively. The singlet–triplet energy splitting (Δ*E*_ST_) allows to predict RISC. In order to evaluate the proximity of the first singlet (S_1_) and first triplet (T_1_) excited states and the possibility of triplet harvesting via RISC, we studied the photophysical properties of the frozen THF solutions of the compounds at 77 K (Figure 7a). The energy values of S_1_ and T_1_ were estimated from the onset of fluorescence and phosphorescence spectra, respectively. The value of Δ*E*_ST_ for compound **QBr2** was found to be 0.53 eV. Such a value is too large to afford efficient singlet–triplet upconversion and confirms that the origin of the emissions is fluorescence peaking at 436 nm and RTP peaking at 553 nm (Figure 7a). Both the donor–acceptor compounds (**QAcr2** and **QPhox2**) demonstrated sufficiently low Δ*E*_ST_ of 0.34 and 0.18 eV, respectively. It is worth noting that the PL intensity maxima of the frozen THF solutions of compounds **QAcr2** and **QPhox2** were considerably shifted (by ca. 120 nm) toward the blue spectral region compared to those observed at room temperature. As it was previously reported,^38^ at room temperature, the movements of the molecules of a solvent rearrange their organization with respect to dipoles of luminophore-optimized electrostatic interaction and lead to stabilization of the excited state of luminophores. Being instantly frozen, molecules of a solvent do not experience such self-organization. This results in less stabilization of the excited state of the dissolved compound.

#### Solid-State Emission Enhancement

**Figure 8:**
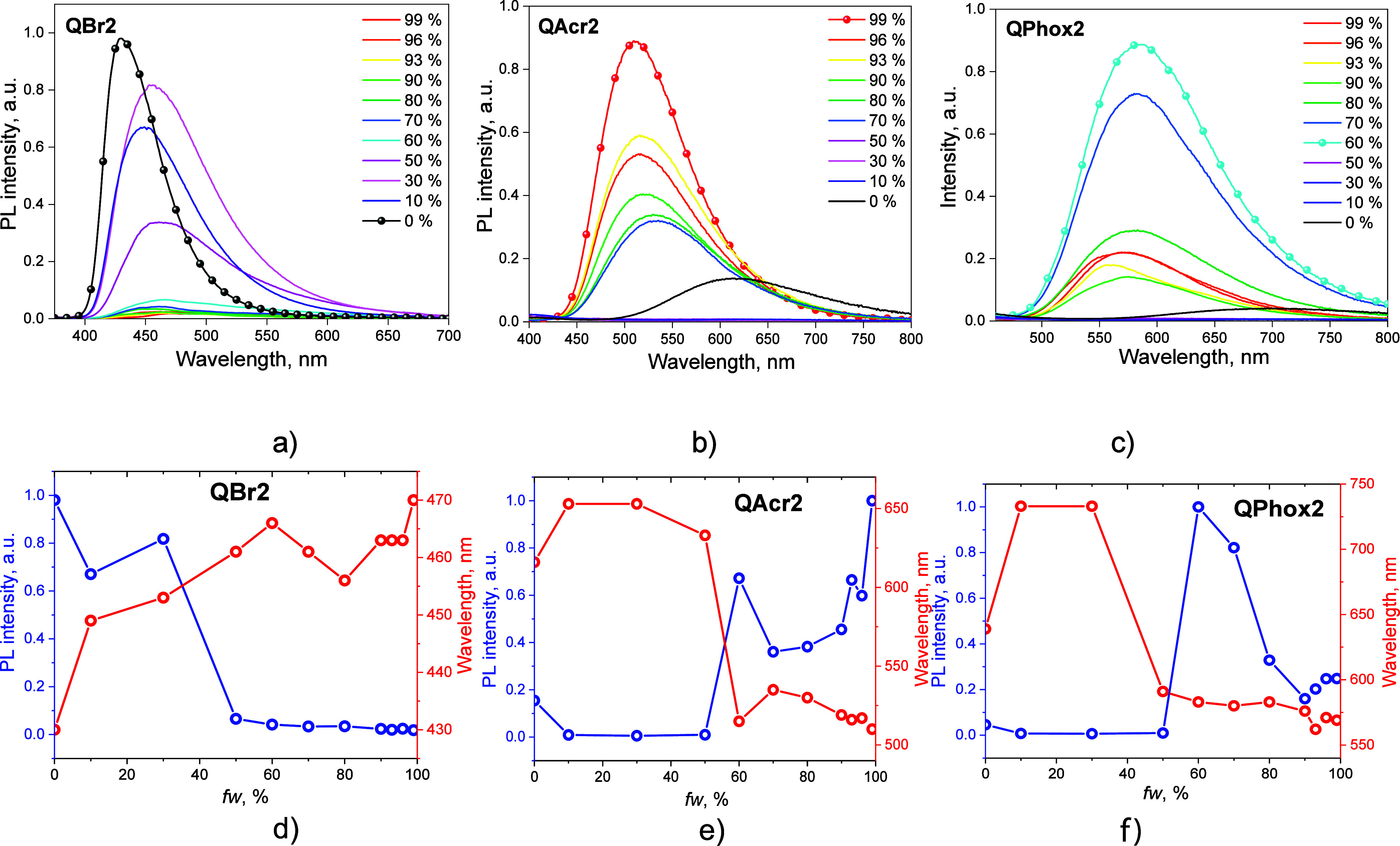
PL spectra of the dispersions of **QBr2** (a), **QAcr2** (b), and **QPhox2** (c) in THF/water mixtures with various *f*_w_. PL intensity and emission wavelength as the function of *f*_w_ for the dispersions of **QBr2** (d), **QAcr2** (e), and **QPhox2** (f) in THF/water mixtures.

In order to investigate whether the differences in PLQY values of the solid films and the dilute solutions of compounds **QBr2**, **QAcr2**, and **QPhox2** are caused by solid-state emission enhancement, we studied the change of photoluminescence intensity of their dispersions in THF/water mixtures with the various volume ratios of THF and water (Figure 8). In the case of bromine-containing compound **QBr2**, 10% of the water fraction (*f*_w_) caused the decrease in PL intensity compared to that observed for the pure THF solution (Figure 8a). The PL maxima bathochromically shifted from 430 to 449 nm due to the increase in the polarity caused by the addition of water (Figure 8d). The further increase in *f*_w_ to 30% resulted in a slight increase in PL intensity, but after increasing the *f*_w_ to 50%, the intensity of emission sharply shrank. The tendency to decline remained until *f*_w_ reached 99%, indicating that the emission of compound **QBr2** in a solid or aggregated state was reduced by aggregation-caused quenching. This observation fully correlates with the higher PLQY of the dilute solution of **QBr2** relative to that of the film. As it was exposed by single-crystal data, in the solid state, molecules of compound **QBr2** experience π–π stacking that noticeably closes the radiative decay channel. The solutions of compounds **QAcr2** and **QPhox2** in THF exhibited a rather weak emission, but after adding water, the PL intensity increased considerably. For the dispersion of compound **QAcr2** (Figure 8b,c), the sharp increase in PL intensity was observed when *f*_w_ reached 60%. Then, the emission intensity decreased when *f*_w_ was raised to 70%. After that point, the PL intensity increased constantly until its maximum *f*_w_ of 99% was reached (Figure 8e,f). The dispersions of compound **QPhox2** exhibited the similar behavior to that of **QAcr2** at low water fractions. The sharp increase in PL intensity was observed at an *f*_w_ of 60%, but afterward, the emission intensity slowly decreased. This observation can be attributed to the formation of aggregates heavy enough to precipitate. The molecules of compounds **QAcr2** and **QPhox2** are of a more propeller-like geometry, while **QBr2** has a more rodlike shape. The propeller-like shape prevents π–π stacking via restriction of intramolecular rotation, thus resulting in higher PLQY of the solid films of compounds **QAcr2** and **QPhox2** compared to those of the solutions.

#### Hosting

The photophysical properties of the molecular dispersions of 1 wt % of the target compounds in the Zeonex polymer matrix were studied. Doping of a small amount of a luminophore into a rigid polymer provides sufficient environmental confinement to minimize molecular motions as well as intermolecular interactions and leads to the stabilization of the triplet excited state. Expectantly, in the presence of oxygen, the molecular mixtures of compounds **QBr2**, **QAcr2**, and **QPhox2** with Zeonex revealed single PL peaks centered at 436, 460, and 485 nm, respectively (Figure 6d–f). The corresponding PL decays were in the nanosecond range, which is characteristic of fluorescence (Figure S7, Table 4, and Table S4). The changes in PL spectra after removal of oxygen from the media disclosed that triplet excited states are depopulated differently in the case of each compound. Considering the molecular mixture with the bromine-containing compound **QBr2**, the emission intensity and the PL decay curve recorded at the emission maximum (436 nm), which closely resembles that observed for the neat film and dilute solutions, did not change upon removal of oxygen. This observation confirms the fluorescent origin of the emission. The additional emission band emerged at 553 nm (τ = 6.1 ms) (Figure 7a, Figure S4, Table 4, and Table S4) It resulted from RTP. The time-resolved measurements allowed to separate the spectrum of RTP **QBr2** using the delay after excitation of 1 ms (Figure 6d). It appears that the presence of heavy bromine atoms in **QBr2** facilitates spin–orbit coupling and promotes the T_1_–S_0_ radiative transition in agreement with the theoretical predictions. The shape of the emission spectrum of acridine-containing compound **QAcr2** doped in Zeonex recorded with a delay of 1 ms was found to be different from its fluorescence spectrum recorded in the presence of oxygen. In addition to the delayed fluorescence band (presumably of TADF) appearing at 460 nm, the shoulder at about 550 nm was observed (Figure 6e). In the PL decay curve recorded at 460 nm, a long-lived component with the lifetime of 56 ms appeared after deoxygenation, which points out to TADF. The band appeared at the similar but slightly redshifted wavelength to that of the fluorescence of **QAcr2**. In addition, the spectrum was broadened with the shoulder at ca. 550 nm (lifetime of 7 ms). It was recognized as RTP since the band is at the significantly redshifted wavelength compared to that of the fluorescence of **QAcr2** (Figure 7a, Figure S4, Table 4, and Table S4). Therefore, it can be concluded that in the case of compound **QAcr2**, triplet excitons are depopulated via two different radiative channels, i.e., via TADF and RTP. However, both processes are very weak and cannot compete with the prompt fluorescence. Phosphorescence was found to be completely suppressed for phenoxazine-containing **QPhox2**. After removal of oxygen, the shape of the PL spectrum remained unchanged; however, its intensity increased by a factor of more than 2. The delayed emission spectra **QPhox2** are the same as the spectrum of fluorescence (Figure 6f). In addition, the delayed emission lifetime of 8.6 ms was obtained from the PL decay curve recorded at the wavelength of analysis of 485 nm. These observations support the TADF of **QPhox2** in agreement with the theoretical predictions (Table 2).

Preparation of the molecular dispersions of emissive compounds into host matrixes still remains the prevalent approach to substantially enhance their TADF. In order to attain efficient Förster resonance energy transfer (FRET), the absorption spectrum of a guest should overlap with the emission of a host.^39^ To avoid energy losses via triplet states of a host, the host should be characterized by a higher T_1_ value than the emitter. Taking this into consideration, we selected *m*CBP as suitable host matrix for compounds **QBr2**, **QAcr2**, and **QPhox2**, owing to its high triplet energy levels (2.84 eV) and deep-blue emission (λ_max_ of ca. 350 nm).^40^ In addition, to gain deeper insight into the origin of emission of compounds **QBr2**, **QAcr2**, and **QPhox2**, we recorded the PL spectra of guest–host systems at the different temperatures. The PL spectra were assigned to the emission of guest compounds, whereas the emission of *m*CBP was not detected. This observation indicates efficient FRET (Figure 7c–e). The emission maxima of the investigated molecular dispersions were slightly blueshifted compared to those observed for the films of pure compounds (Figure S6) due to the influence of the polarity of the host. Photoluminescence decay curves are shown in Figure S8. For compound **QBr2**, doping into *m*CBP resulted in the appearance of a shoulder at ca. 527 nm (Figure 7c). Taking into account the long radiative decay of the band (τ = 6.45 ms) and having in mind that the similar band with the intensity maximum at 553 nm emerged for a 1 wt % molecular dispersion of **QBr2** in Zeonex after the removal of oxygen, we suspected that its origin is RTP. The study of photoluminescence intensity as a function of temperature revealed significant shrinkage of the shoulder intensity, confirming that the emission originates from the triplet excited state. The intensity of the major peak (433 nm) was not influenced by the changes in the temperature, which is evident for fluorescence. In contrast, the intensities of emission of compounds **QAcr2** and **QPhox2** molecularly dispersed in *m*CBP increased upon heating, pointing out to the thermal activation of the processes. The corresponding PL maxima were not shifted with a change in the temperature. The examination of the decay profiles of the molecular dispersions of the compounds revealed the gradual decrease in the delayed components with the increase in the temperature (Figure S8). The molecular dispersions of the compounds in *m*CBP afforded much smaller singlet–triplet energy splittings compared to those estimated for the frozen THF solutions (Figure 7f and Table 4). They were calculated to be 0.17 and 0.08 eV for the dispersions containing compounds **QAcr2** and **QBr2**, respectively. Therefore, it can be stated that the origin of emission of 10 wt % molecular dispersions of compounds **QAcr2** and **QPhox2** in *m*CBP is mostly TADF. It should be noted that at 77 K, the molecular dispersion of **QAcr2** in *m*CBP is characterized by a PL spectrum with two bands peaking at 486 and 524 nm. Those bands are related to fluorescence and phosphorescence, respectively. Practically the same shape of the PL spectrum of the solution, film, or molecular dispersion in *m*CBP of **QAcr2** was recorded at room temperature. It had the main maximum at 493 nm and the shoulder at 514 nm. This observation suggests the combination of TADF and RTP of **QAcr2** molecularly dispersed in mCBP similarly to that observed for the molecular dispersion in Zeonex (Figures 6e and 7d).

### Electroluminescence

**Figure 9:**
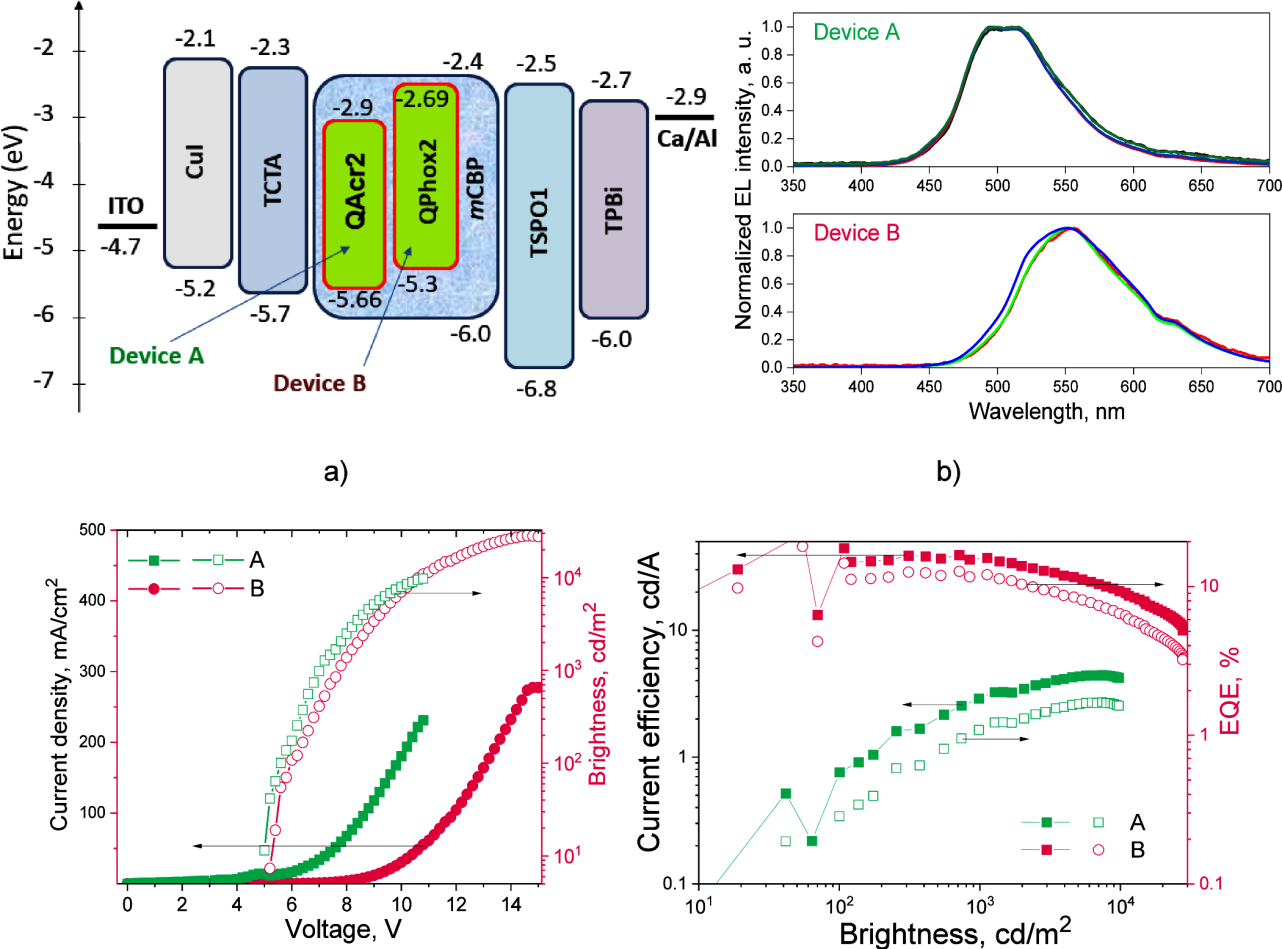
Equilibrium energy diagram (a), EL spectra (b) recorded at the different voltages, plots of current density and brightness vs voltage (c), and current efficiency and EQE vs brightness of devices A and B.

The electroluminescent (EL) properties of **QAcr2** and **QPhox2** were studied using the device structure ITO/CuI(7 nm)/TCTA(40 nm)/light-emitting layer(25 nm)/TSPO1(10 nm)/TPBi(20 nm)/Ca/Al. The ITO acted as an anode material. Keeping in mind the values of the ionization potential of **QAcr2** (5.66 eV) and **QPhox2** (5.3 eV) (Figure 5f), copper iodide (CuI) and 4,4′,4-tris(carbazol-9-yl)triphenylamine (TCTA) were selected for ensuring good injection and transport of holes.^41−43^ Taking into account the values of the electron affinities of **QAcr2** (2.9 eV) and **QPhox2** (2.69 eV) (Figure 5f), diphenyl[4-(triphenylsilyl)phenyl]phosphine oxide (TSPO1), 2,2′,2″-(1,3,5-benzenetriyl)-tris(1-phenyl-1*H*-benzimidazole) (TPBi) and calcium (Ca) were selected for ensuring good injection and transport of electrons.^44,45^ According to the equilibrium energy diagram (Figure 9a), the hole–electron recombination zone should be located in the light-emitting layer. The layers of codeposited **QAcr2** or **QPhox2** (10 wt %) and *m*CBP were utilized as the light-emitting layers of devices A and B, respectively. Because of the high singlet and triplet energy levels of TCTA, *m*CBP, and TSPO1, there were no obvious channels for losing electrically generated excitons except via the emitters **QAcr2** or **QPhox2**. Triplets could be harvested to electroluminescence because of the TADF of **QAcr2** or **QPhox2**. Indeed, the EL spectra well reflected the corresponding PL spectra of **QAcr2** and **QPhox2** (Figures 7d and 9b). No changes in the colors of the EL of devices A and B were observed after the change of the voltage. The EL spectra recorded at different voltages were practically the same (Figure 9b).

EL with a brightness of ca. 10 cd/m^2^ was observed at the turn-on voltage of 5 V (Figure 9c). The brightness of devices A and B exceeded 5000 cd m^–2^ that covers requirements for OLED display applications.^46^ Devices A and B exhibited very different maximum EQEs of 1.66 and 12.3%, respectively (Figure 9d). The maximum efficiencies of device A were observed at high brightnesses apparently because of low and unbalanced charge carrier mobilities of **QAcr2** (Figure 5b). Since the PLQY values of solid-state samples of **QAcr2** and **QPhox2** are close, the different EQE values of devices A and B should be attributed to the different triplet harvesting abilities and charge-transporting properties. The hole and electron mobilities of 3.2 × 10^–4^ and 1.5 × 10^–4^ cm^2^ V^–1^ s^–1^, respectively, were observed for the layer of **QPhox2** at an electric field of 5.8 × 10^5^ V/cm. At a considerably higher electric field of 2.5 × 10^6^ V/cm, hole and electron mobilities of **QAcr2** reached only 2.7 × 10^–5^ and 3.5 × 10^–6^ cm^2^ V^–1^ s^–1^, respectively (Figure 5b). In contrast to the conventional TADF of **QPhox2**, the solid-state samples of **QAcr2** showed dual long-lived emissions, i.e., TADF and RTP. It can be concluded that the RTP of **QAcr2** dramatically reduces EQEs of the device. The emissive relaxation of triplets is much slower than the emissive relaxation of singlets. This result is in good agreement with the previously reported information on OLED emitters, which showed the combination of TADF and RTP.^34,47,48^

## Conclusions

Three new donor–acceptor–donor-type organic semiconductors based on the newly developed rodlike electron acceptor benzodioxinoquinoxaline were designed and synthesized in high yields, employing microwave irradiation-assisted nucleophilic aromatic substitution and Buchwald–Hartwig cross-coupling reactions. The compounds were characterized by high thermal stability with 5% weight loss temperatures ranging from 330 to 460 °C. For bromine-substituted benzodioxinoquinoxaline, transformation into a glassy state was detected at 158 °C. Three different types of emissions were observed for the compounds and supported by quantum chemical calculations. Both acridine- and phenoxazine-containing compounds demonstrated bipolar charge transport. The phenoxazine-containing compound showed respective hole and electron mobility values of 3.2 × 10^–4^ and 1.5 × 10^–4^ cm^2^ V^–1^ s^–1^ at the electric field of 5.8 × 10^5^ V/cm. The electroluminescent device with a light-emitting layer based on the benzodioxinoquinoxaline derivative containing phenoxazine moieties demonstrated the maximum external quantum efficiency of 12.3%.

## Supporting Information Available

Computational details and theoretical background, details of synthesis and identifications of compounds, corresponding ^1^H NMR spectra, crystal data and structure refinements of compound **QBr2**, TOF signals and details of thickness measurements of TOF samples, TGA and DSC curves of compounds **QBr2** and **QPhox2**, and additional photophysical data (PDF)
