# Supplementary material for: Efficient Microwave Irradiation-Assisted Synthesis of Benzodioxinoquinoxaline and Its Donor-Variegated Derivatives Enabling Long-Lived Emission and Efficient Bipolar Charge Carrier Transport
Source: ACS Mater Au. 2024 Aug 14;4(6):628–42. doi: 10.1021/acsmaterialsau.4c00050 (PMC11565285; doi:10.1021/acsmaterialsau.4c00050)
Supplement: Supplementary file 1 — mg4c00050_si_001.pdf [file mg4c00050_si_001.pdf]

Supporting Information

Efficient Microwave Irradiation-Assisted Synthesis of Benzodioxinoquinoxaline and Its Donor-Variegated Derivatives Enabling Long-Lived Emission and Efficient Bipolar Charge Carrier Transport

Liliia Deva[1,2**], Mariia Stanitska[2,3**], Levani Skhirtladze[2,4], Amjad Ali[4], Glib Baryshnikov*[4], Dmytro Volyniuk[2], Stepan Kutsiy[1], Mykola Obushak[3], Monika Cekaviciute[2], Pavlo Stakhira[1], Juozas Vidas Grazulevicius*[2]

[1]*Department of Electronic Engineering, Institute of Telecommunications, Radioelectronics and Electronic Engineering, Lviv Polytechnic National University, Stepan Bandera st. 12, 79013 Lviv, Ukraine*

[2]*Kaunas University of Technology, Baršausko 59, 51423 Kaunas, Lithuania. *email: juozas.grazulevicius@ktu.lt;*

[3]*Ivan Franko National University of Lviv, Kyryla i Mefodiya 6, Lviv, Ukraine*

[4] *Laboratory of Organic Electronics, Department of Science and Technology, Linköping University, Norrköping SE-60174, Sweden. *email: glib.baryshnikov@liu.se*

**Lilia Deva and Mariia Stanitska share the equal contribution to this article

1. Computational Detials and Theoretical Background

1.1 Computational details

The molecular structures of the designed compounds were optimized in the ground state (S_0) at the density functional theory (DFT) level in the toluene and benzene as solvents using the B3LYP[1,2] functional and 6-31G(d)[3,4] basis set. The first excited singlet (S_1) and triplet (T_1) states were optimized using the range-separated functional CAM-B3LYP by Handy[5] with 6-31G(d) basis set employing TD-

DFT with the Tamm-Dancoff approximation (TDA)[6]. We checked the vibrational frequencies for both ground and excited state optimizations and all of them were positive, which means all of them were real. The Polarizable Continuum Model (PCM)[7] was used to account for the solvent effect for S_0, S_1 and T_1 states. The gap-tuned range-separated LC-ωPBE[8] functional with the 6-31G(d)[3,4,9] basis set within the TDA formalism was used to compute the fluorescence (λ_{flu}), phosphorescence (λ_{phos}), and using the single point energy calculation of the S_1 and T_1 optimized chemical structures of all three compounds. We, henceforth, symbolise the LC-ωPBE with regulated ω value as LC-ω*PBE. We followed the procedures for ω tuning proposed somewhere else[10,11]. The Gaussian 16[12] software was used to perform the up-mentioned calculations in toluene and benzene solvents.

1.2 Spin-orbit coupling (SOC)

Based on the S_1 and T_1 state optimized geometry of **QBr2, QAcr2,** and **QPhox2**, the spin-orbit coupling (SOC) effect was treated as a perturbation based on the scalar relativistic (SR) orbitals after SCF and TDA-DFT computations (pSOC-TDADFT)[13] with PBE0[14,15] functional and TZP basis set[16] in benzene as a solvent using the ADF software (version 2021.102)[17]. The intersystem crossing (ISC) and reverse intersystem crossing (RISC) rates were predicted using semi-classical Marcus theory[18]. The k_{ISC} and k_{RISC} were calculated in benzene solvent. The SOC matrix elements $\langle S_1|\hat{H}_{SO}|T_1\rangle$ were calculated as root mean squares at S_1 state geometry, i.e. as square root of the sum of squares of spin-orbit coupling matrix elements of all triplet state sublevels (m=0,±1) of the uncoupled states:

$$\langle S_1 \,|\, \hat{H}_{SO} \,|\, T_1\rangle = \sqrt{\sum_{m=0,\pm1} \langle S_1 \,|\, \hat{H}_{SO} \,|\, T_1^m\rangle^2} \qquad (1)$$

The spin-orbit coupling operator $\hat{H}_{SO}$ was considered in our calculations within the zeroth-order regular approximation (ZORA).[19] The fluorescence rate constant (k_{flu}) for all molecules was predicted according to the following relationship:

$$k_{flu} = \frac{1}{\tau_{av}} = \frac{2(\Delta E^2)f}{c^3} \qquad (2)$$

where ΔE and f – the energy and intensity of the corresponding singlet-singlet transition – were calculated at the S_1 state geometry with account of SOC perturbations.

The average phosphorescence lifetime (τ_{av}) for T_1^m sublevels for all molecules was estimated using the following averaging formula:

$$\tau_{av(phos)} = \frac{3}{(\frac{1}{\tau_x}+\frac{1}{\tau_y}+\frac{1}{\tau_z})} \quad (3)$$

Based on *τav* value the averaged phosphorescence rate constant (*k_{phos}*) was estimated following the inverse τ_{av} = 1/k_{phos} relation.

The rates of intersystem crossing (ISC) and reverse intersystem crossing (RISC) were estimated using semiclassical Marcus theory expression:

$$k_{(R)ISC} = \frac{2\pi}{\hbar}\langle S_1 \,|\, \hat{H}_{SO} \,|\, T_1\rangle^2 \sqrt{\frac{1}{4\pi k_b T\lambda}} exp[-\frac{(\Delta E_{ST}+\lambda)^2}{4k_b T\lambda}] \quad (4)$$

where k_b is the Boltzmann constant, T is the temperature, which is set to 298 K, ΔE_{ST} is adiabatic excitation energy difference, λ is the reorganization energy induced by S_1-T_1 ISC or T_1-S_1 RICS. ΔE_{ST} < 0 for the k_{ISC} rate, while for RISC ΔE_{ST} is negative with the same magnitude (i.e. ΔE_{ST} > 0). Similarly, the different reorganization energies were used for k_{ISC} and k_{RISC} named as λ_T and λ_S, respectively:

$$\lambda_T = E(T_1)^{S_1\,geom} - E(T_1)^{T_1\,geom} \quad (5)$$

$$\lambda_S = E(S_1)^{T_1\,geom} - E(S_1)^{S_1\,geom} \quad (6)$$

2. Materials:

Synthesis of 2,3-bis(4-bromophenyl)-6,7-difluoroquinoxaline QF2Br2:

4,5-Difluorobenzene-1,2-diamine (2.54 g, 17.6 mmol) and 1,2-bis(4-bromophenyl)ethane-1,2-dione (5 g, 13.5 mmol) were dissolved in glacial acetic acid (100 mL) and refluxed at 110 °C for 24 h. After cooling down to room temperature, the mixture was poured into cold water and filtered off on a glass filter. The crude product was purified by column chromatography on silica gel using hexane/dichloromethane with volume ratio 4:1 as the eluent to obtain a white solid, which was recrystallized to afford pure product.

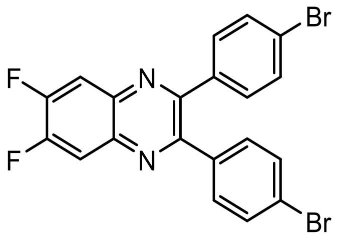

Yield 92%; white solid; M = 476.12

^{1}H NMR (400 MHz, $CDCl_3$) δ 7.89 (t, *J* = 9.2 Hz, 2H), 7.51 (d, *J* = 8.1 Hz, 4H), 7.38 (d, *J* = 8.1 Hz, 4H)

^{13}C NMR (101 MHz, $CDCl_3$) δ 154.30, 154.12, 152.29, 151.72, 151.54, 138.78, 138.72, 138.66, 137.23, 131.93, 131.50, 124.21, 115.04, 114.97, 114.91, 114.85

^{1}H NMR specrum of 2,3-bis(4-bromophenyl)-6,7-difluoroquinoxaline QF2Br2:

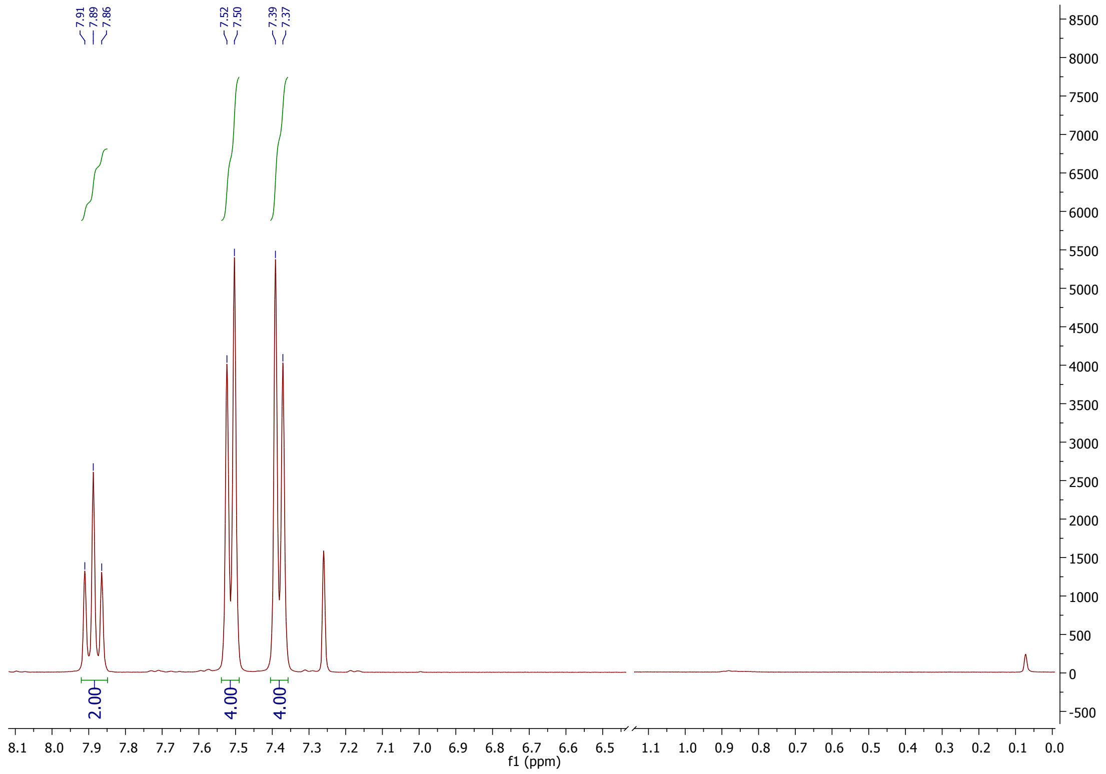

^{13}C NMR spectrum of 2,3-bis(4-bromophenyl)-6,7-difluoroquinoxaline QF2Br2:

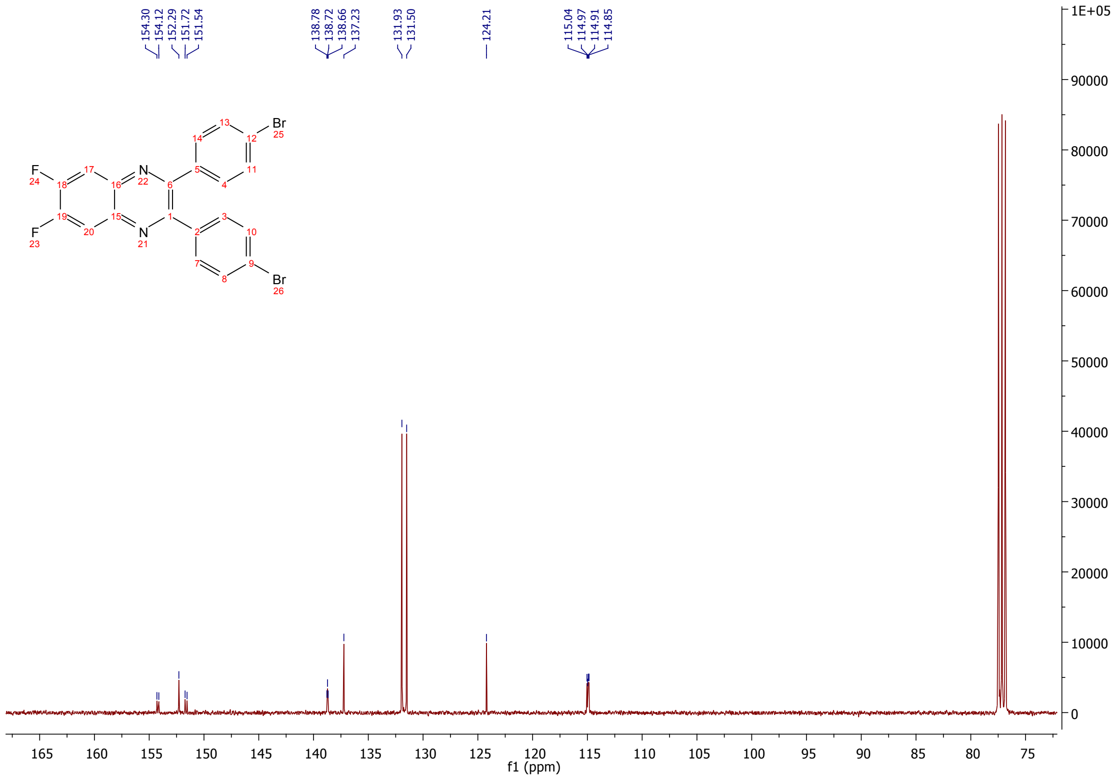

Synthesis of 2,3-bis(4-bromophenyl)benzo[5,6][1,4]dioxino[2,3-g]quinoxaline QBr2:

A mixture of 2,3-bis(4-bromophenyl)-6,7-difluoroquinoxaline **QF2Br2** (1 g, 2.1 mmol), pyrocatechol (0.46 g, 4.2 mmol), potassium carbonate (0.87g, 6.3 mmol) and dry dimethylformamide (15 mL) was loaded in a G30 reaction vial under argon atmosphere. The vial with reaction mixture was put in the microwave at 100°C for 30 minutes. When the reaction was completed, mixture was poured into cold water and precipitate was separeted by vacuum filtration. The product was washed with water and dried.

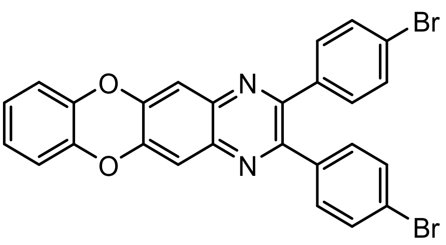

Yield 91%; yellowish powder; M = 546.22

^{1}H NMR (400 MHz, $CDCl_3$) δ 7.53 – 7.44 (m, 6H), 7.36 (d, *J* = 8.2 Hz, 4H), 7.00 (s, 4H)

^{13}C NMR (101 MHz, $CDCl_3$) δ 151.03, 145.49, 140.90, 139.77, 137.75, 131.80, 131.49, 124.62, 123.68, 116.83, 112.85

MS (ESI), m/z: 546.76 [M^+]

FT-IR (KBr) cm^{-1}: 3042 (Ar C-H); 1484, 1476, (Ar C=C); 1390, 1350, (Ar C-N); 1269, 1206 (C-O-C)

^{1}H NMR spectrum of of 2,3-bis(4-bromophenyl)benzo[5,6][1,4]dioxino[2,3-g]quinoxaline QBr2:

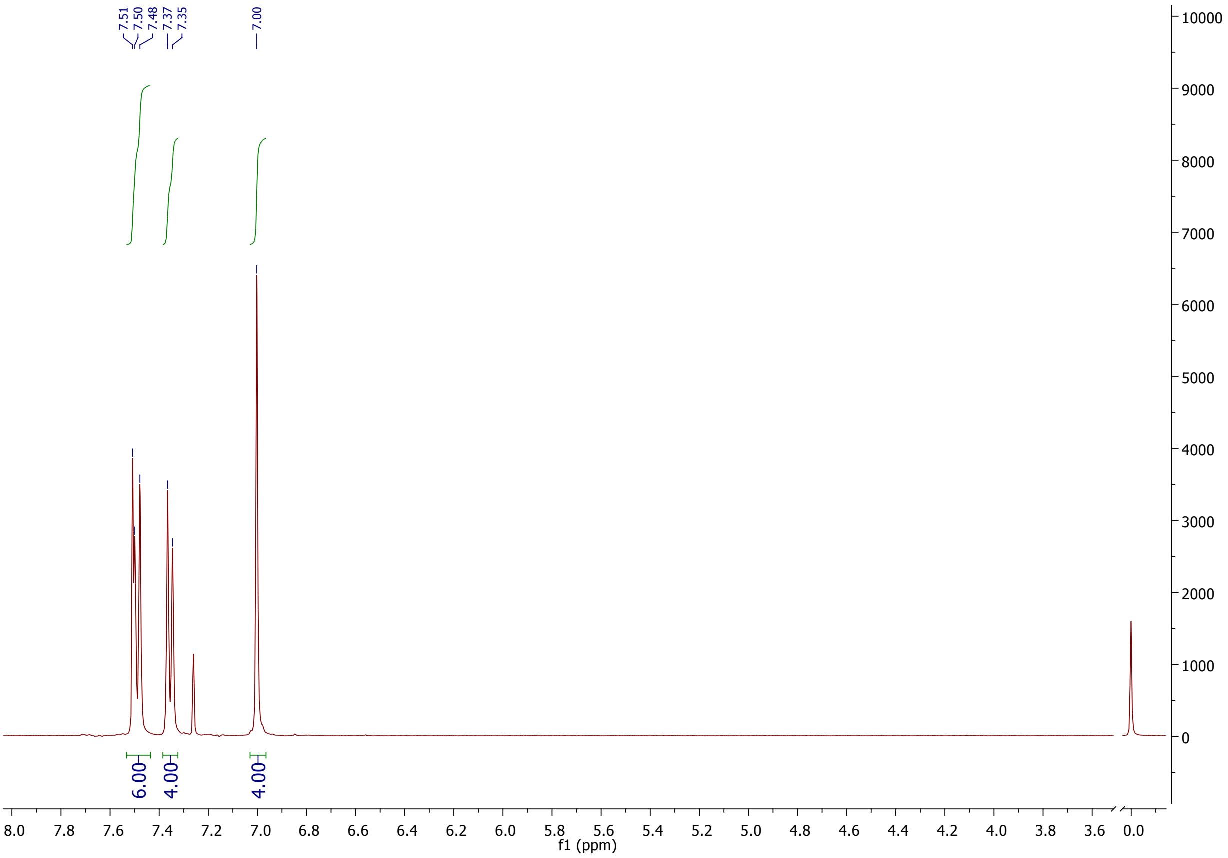

^{13}C NMR spectrum of 2,3-bis(4-bromophenyl)benzo[5,6][1,4]dioxino[2,3-g]quinoxaline QBr2:

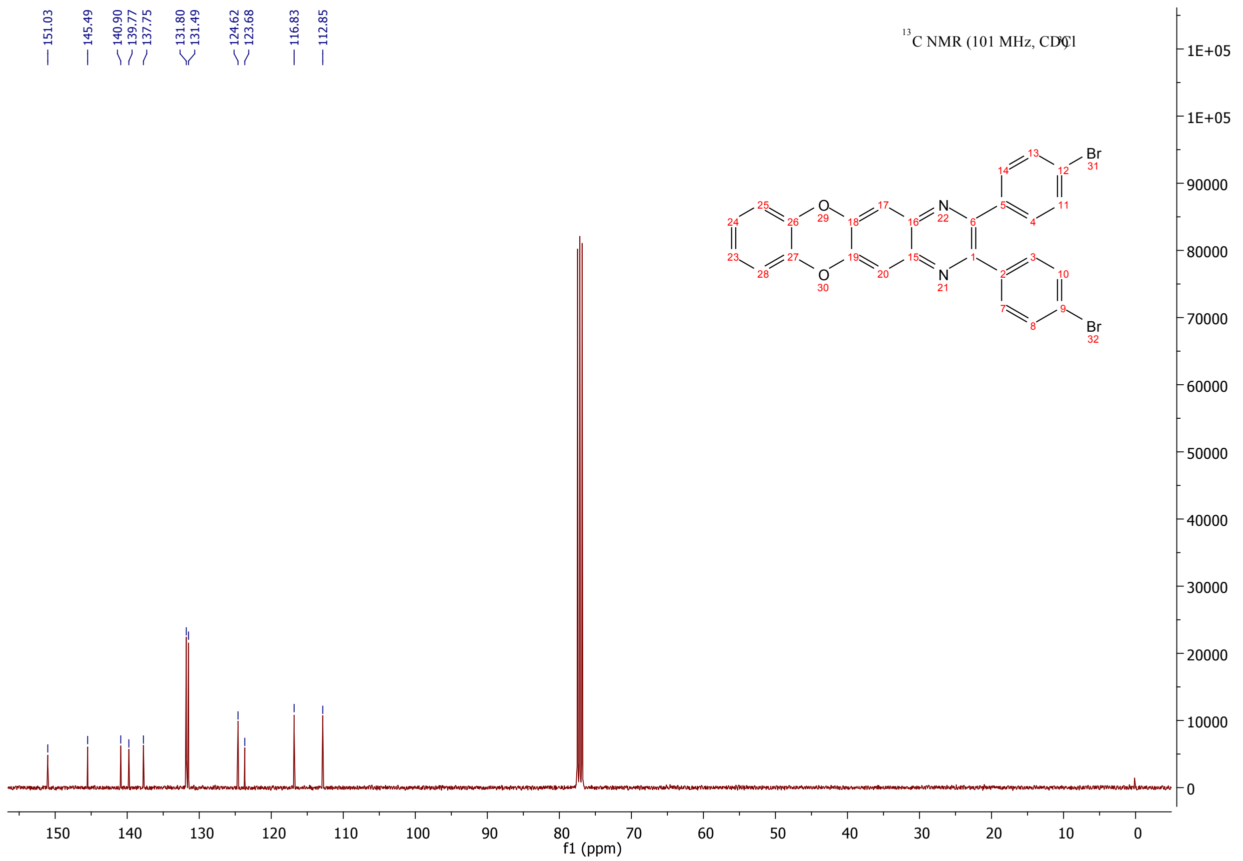

Synthesis of 10,10'-((6,7-difluoroquinoxaline-2,3-diyl)bis(4,1-phenylene))bis(9,9-dimethyl-9,10-dihydroacridine) QF2Acr2:

A mixture of 2,3-bis(4-bromophenyl)-6,7-difluoroquinoxaline **QF2Br2** (0.6 g, 1.2 mmol), 9,9-dimethyl-9,10-dihydroacridine (0.5 g, 2.78 mmol), tris(dibenzylideneacetone) dipalladium (0) (0.05 g, 0.06 mmol), X-phos (0.04 g, 0.08 mmol), sodium tert-butoxide (0.12 g, 1.29 mmol) and dry toluene (15 mL) was loaded in a G30 reaction vial under argon atmosphere. The vial with reaction mixture was put in the microwave at 130°C for 30 minutes. When the reaction was completed, crude product was purified by column chromatography on silica gel using hexane/ethylacetate in volume ratio 4:1 as an eluent.

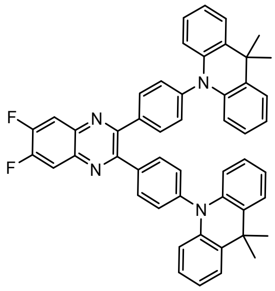

Yield 86%; yellow powder; M = 732.88

^1H NMR (400 MHz, $CDCl_3$) δ 8.03 (t, *J* = 9.2 Hz, 2H), 7.83 (d, *J* = 8.2 Hz, 4H), 7.43 (dd, *J* = 14.0, 7.9 Hz, 8H), 6.87 (dt, *J* = 20.7, 7.3 Hz, 8H), 6.32 (d, *J* = 8.0 Hz, 4H), 1.69 (s, 12H).

^{13}C NMR (101 MHz, $CDCl_3$) δ 153.31, 142.54, 140.74, 138.31, 132.55, 131.59, 130.43, 126.65, 125.28, 121.01, 114.14, 36.16, 31.03, 29.85.

^{1}H NMR spectrum of 10,10'-((6,7-difluoroquinoxaline-2,3-diyl)bis(4,1-phenylene))bis(9,9-dimethyl-9,10-dihydroacridine) QF2Acr2:

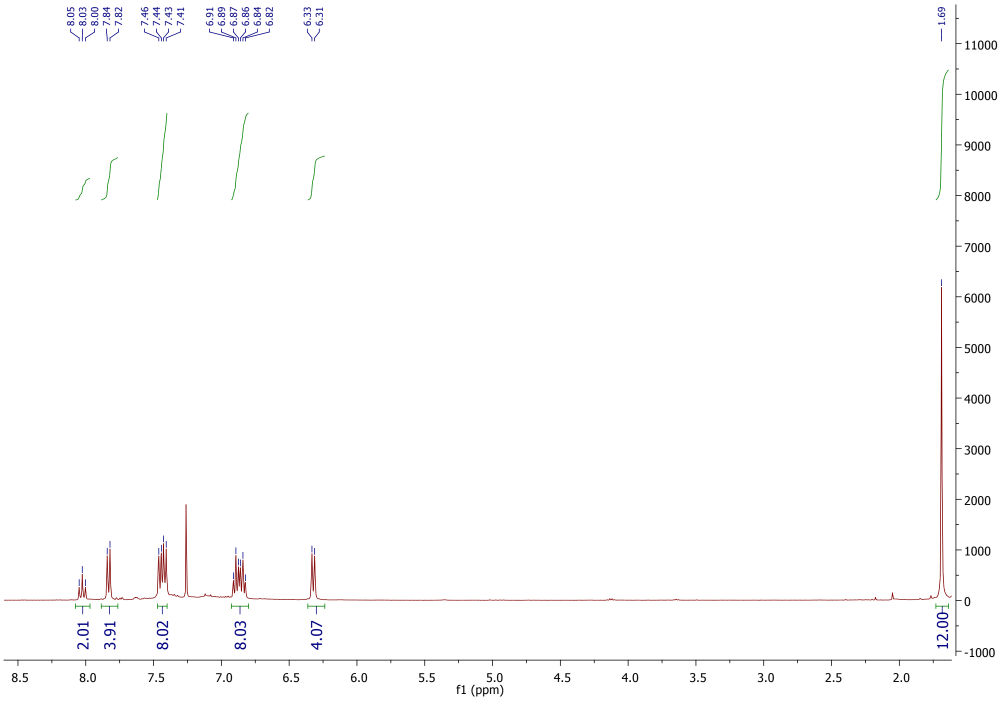

^{13}C NMR spectrum of 10,10'-((6,7-difluoroquinoxaline-2,3-diyl)bis(4,1-phenylene))bis(9,9-dimethyl-9,10-dihydroacridine) QF2Acr2:

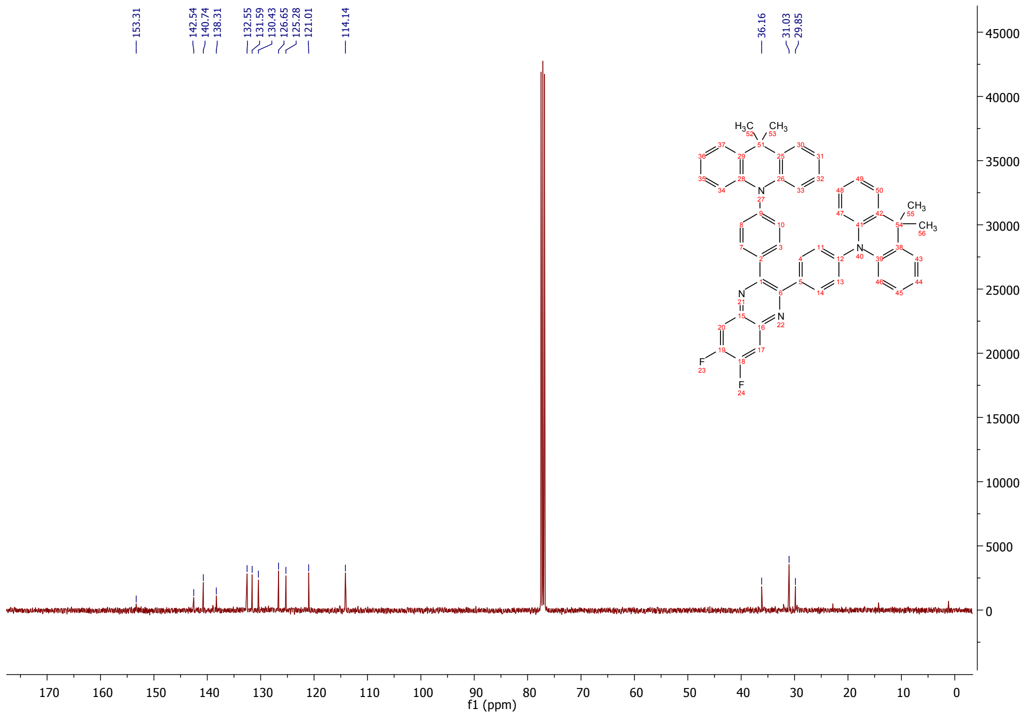

Synthesis of 2,3-bis(4-(9,9-dimethylacridin-10(9H)-yl)phenyl)benzo[5,6][1,4]dioxino[2,3-g]quinoxaline QAcr2:

A mixture of 10,10'-((6,7-difluoroquinoxaline-2,3-diyl)bis(4,1-phenylene))bis(9,9-dimethyl-9,10-dihydroacridine) **QF2Acr2** (0.2 g, 0.27 mmol), pyrocatechol (0.06 g, 5.45 mmol), potassium carbonate (0.11 , 0.81 mmol) and dry dimethylformamide (15 mL) was added in a G30 reaction vial under argon atmosphere. The vial with reaction mixture was put in the microwave at 100°C for 30 minutes. When the reaction was completed, mixture was poured into water and precipitate was separated by vacuum filtration. The solid was washed with water and dried.

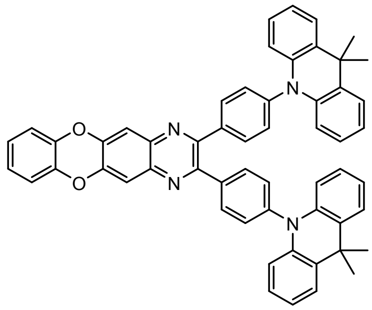

Yield 91%, yellow powder; M = 802.98

^{1}H NMR (400 MHz, $CDCl_3$) δ 7.81 (d, *J* = 8.1 Hz, 4H), 7.65 (s, 2H), 7.45 (d, *J* = 7.5 Hz, 4H), 7.39 (d, *J* = 8.1 Hz, 4H), 7.05 (s, 4H), 6.86 (dt, *J* = 15.1, 7.1 Hz, 8H), 6.33 (d, *J* = 7.9 Hz, 4H), 1.69 (s, 12H).

^{13}C NMR (101 MHz, $CDCl_3$) δ 162.99, 152.36, 145.94, 142.37, 141.26, 141.11, 140.28, 139.18, 132.85, 131.79, 130.64, 126.96, 125.53, 124.99, 121.22, 117.20, 114.47, 113.35, 36.46, 31.35.

MS (ESI), m/z: 803.11 [M^+]

FT-IR (KBr) cm^{-1}: 3030 (Ar C-H); 2965, 2954 (Alk C-H); 1498, 1483, (Ar C=C); 1355, 1330, (Ar C-N); 1266, 1204 (C-O-C)

^{1}H NMR spectrum of 2,3-bis(4-(9,9-dimethylacridin-10(9H)-yl)phenyl)benzo[5,6][1,4]dioxino[2,3-g]quinoxaline QAcr2:

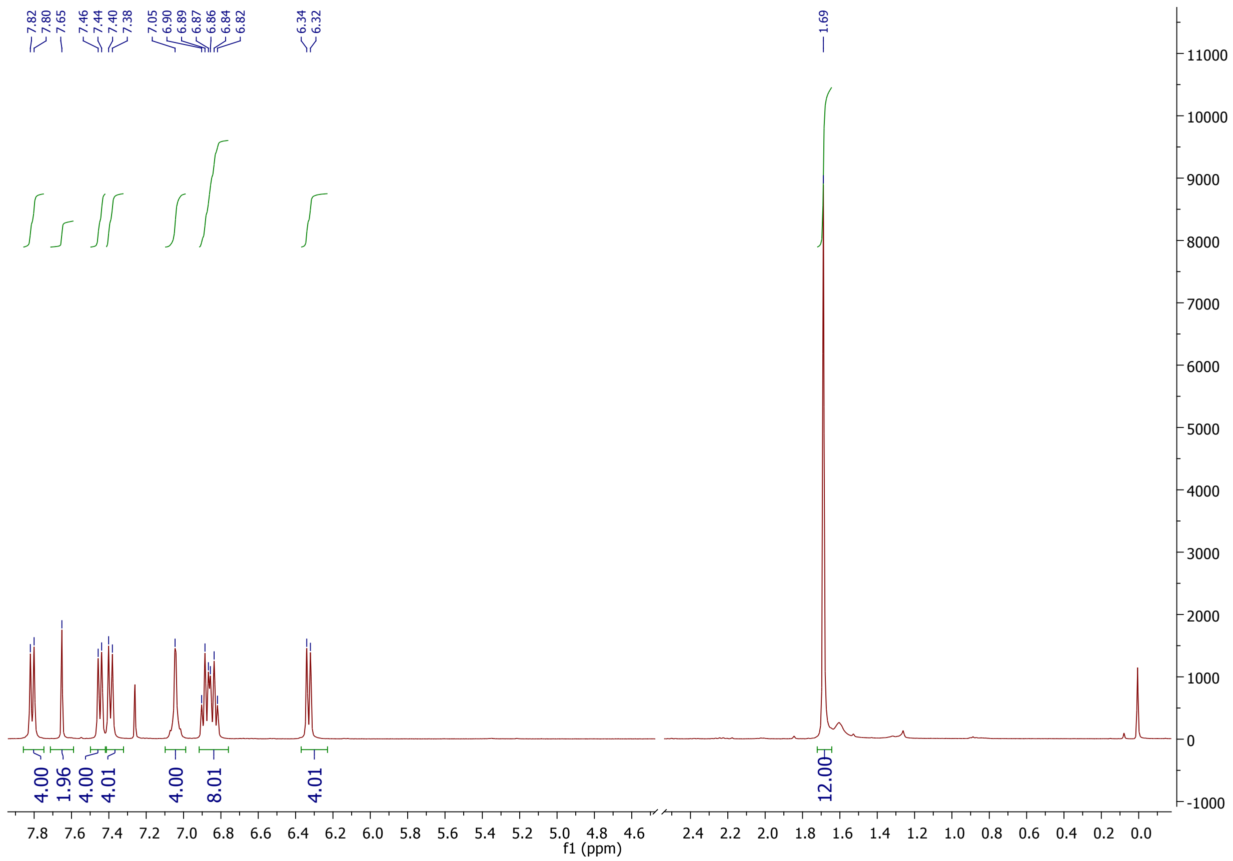

^{13}C NMR spectrum of 2,3-bis(4-(9,9-dimethylacridin-10(9H)-

yl)phenyl)benzo[5,6][1,4]dioxino[2,3-g]quinoxaline QAcr2:

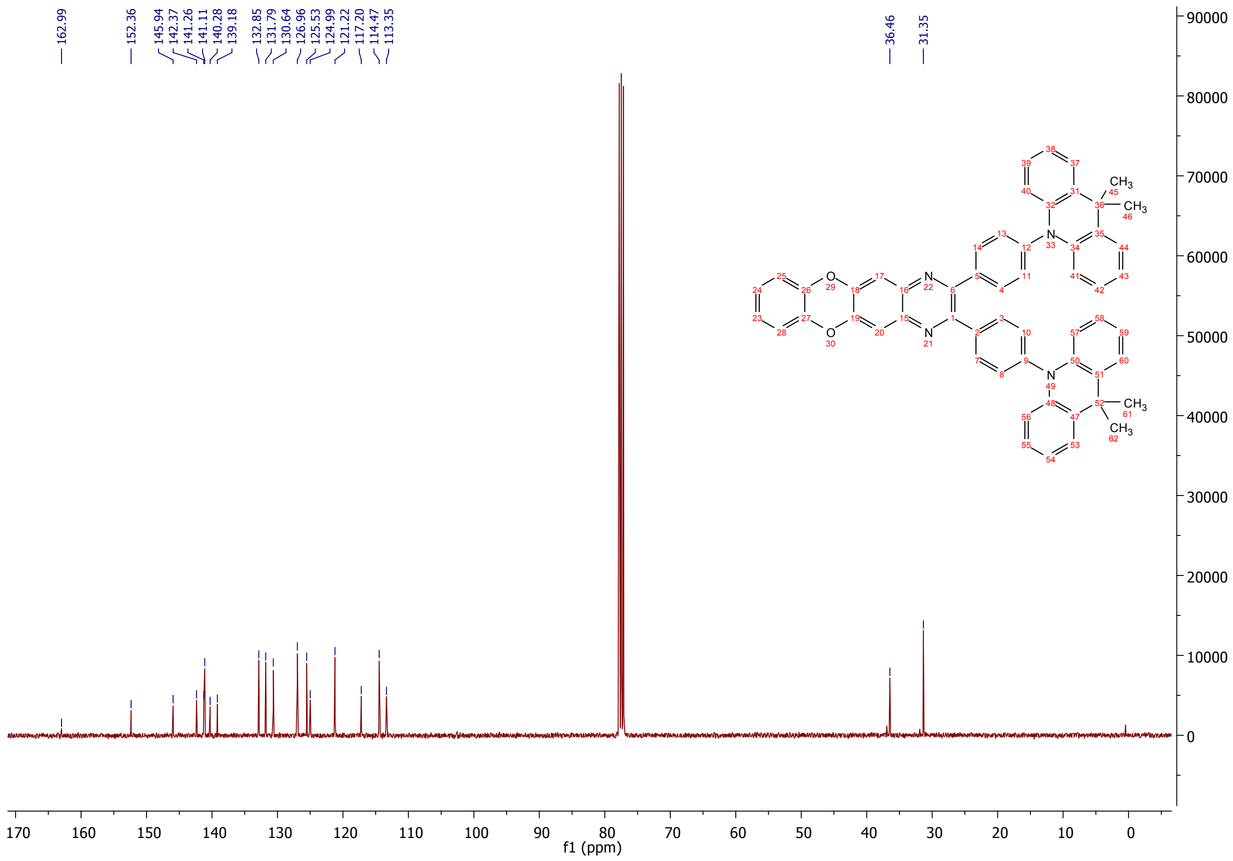

Synthesis of 2,3-bis(4-(10H-phenoxazin-10-yl)phenyl)benzo[5,6][1,4]dioxino[2,3-g]quinoxaline QPhox2:

A mixture of 2,3-bis(4-bromophenyl)benzo[5,6][1,4]dioxino[2,3-g]quinoxaline **QBr2** (0.3 g, 0.54 mmol), 10*H*-phenoxazine (0.52 g, 1.2 mmol), tris(dibenzylideneacetone) dipalladium (0) (0.02 g, 0.02 mmol), X-phos (0.01 g, 0.03 mmol), sodium tert-butoxide (0.11 g, 1.2 mmol) and dry toluene (15 mL) was added in a G30 reaction vial under argon atmosphere. The vial with reaction mixture was put in the microwave at 130°C for 30 minutes. When the reaction was completed, crude product was purified by column chromatography on silica gel using hexane/ethylacetate with volume ratio 4:1 as an eluent.

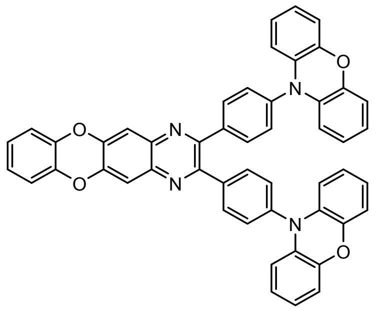

Yield 73%, orange powder; M = 750.81

^{1}H NMR (400 MHz, $CDCl_3$) δ 7.74 (d, *J* = 8.1 Hz, 4H), 7.62 (s, 2H), 7.37 (d, *J* = 8.1 Hz, 4H), 7.04 (s, 4H), 6.69 (d, *J* = 7.8 Hz, 4H), 6.62 (t, *J* = 7.6 Hz, 4H), 6.51 (t, *J* = 7.6 Hz, 4H), 5.95 (d, *J* = 7.9 Hz, 4H).

^{13}C NMR (101 MHz, $CDCl_3$) δ 151.70, 145.69, 144.03, 140.90, 139.95, 139.81, 139.12, 134.07, 132.68, 130.98, 124.70, 123.54, 121.70, 116.88, 115.69, 113.27, 112.99.

MS (ESI), m/z: 750.5 [M^+]

FT-IR (KBr) cm^{-1}: 3060 (Ar C-H); 1491, 1487, (Ar C=C); 1357, 1332, 1321, (Ar C-N); 1291, 1268 (C-O-C)

1H NMR spectrum of 2,3-bis(4-(10H-phenoxazin-10-yl)phenyl)benzo[5,6][1,4]dioxino[2,3-g]quinoxaline QPhox2:

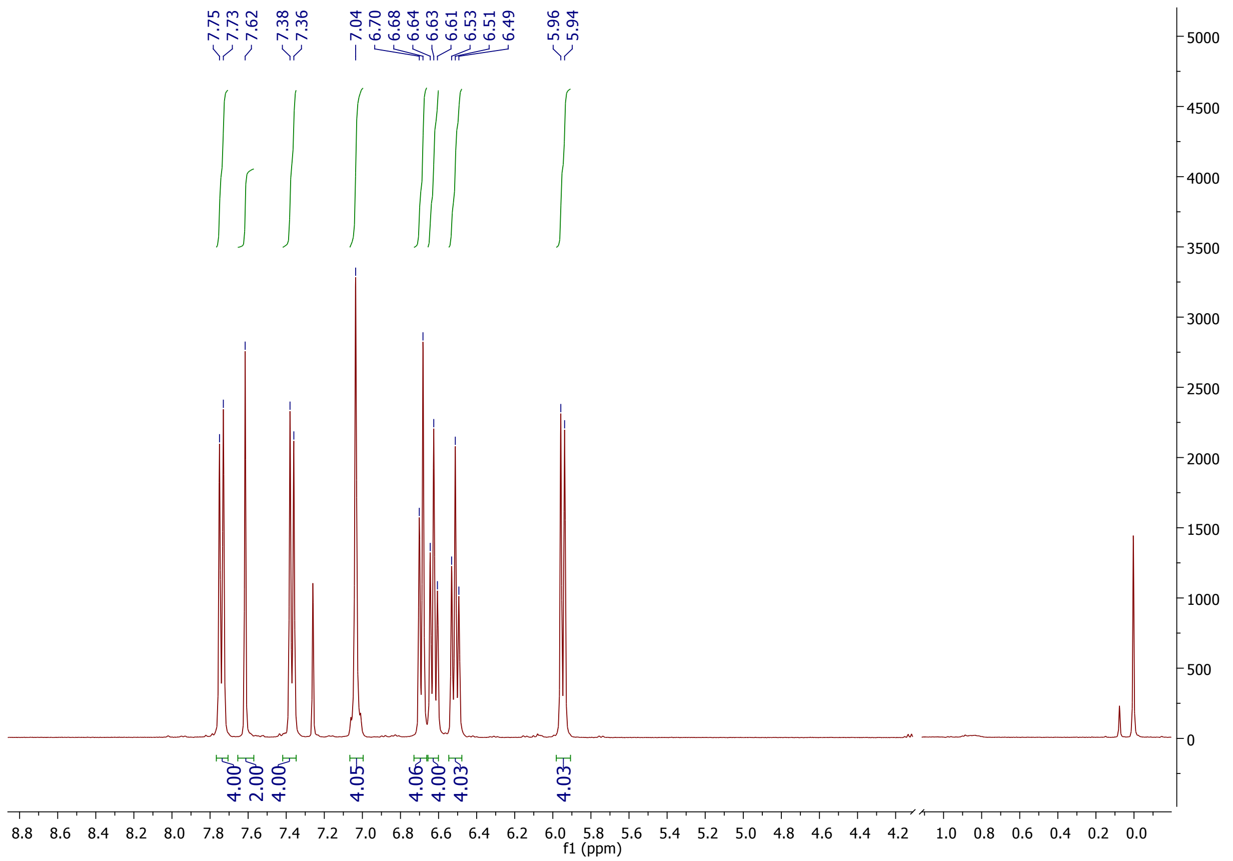

^{13}C NMR spectrum of 2,3-bis(4-(10H-phenoxazin-10-yl)phenyl)benzo[5,6][1,4]dioxino[2,3-g]quinoxaline QPhox2:

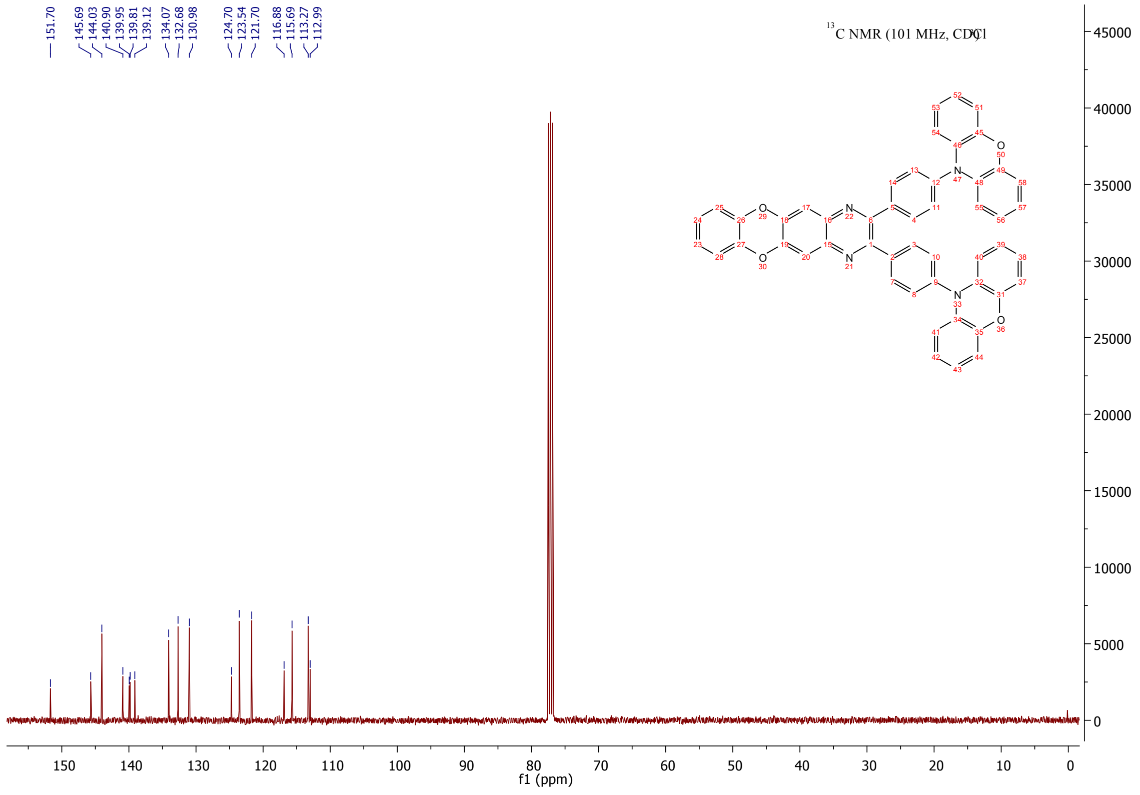

3. Tables and Figures

Table S1. Crystal data and structure refinements of compound **QBr2**.

**Formula**	$C_{26}H_{14}Br_2N_2O_2$
**Molecular weight**	546.22
**Crystal system**	triclinic
**Space group**	P -1
**a (Å)**	8.1659 (11)
**b (Å)**	10.1207 (19)
**c (Å)**	13.3601 (18)
α **(deg)**	74.453 (16)
β **(deg)**	89.305 (11)
γ **(deg)**	89.586 (14)
**Cell volume, (Å^3)**	1063.65
**Z, Z'**	5; 0

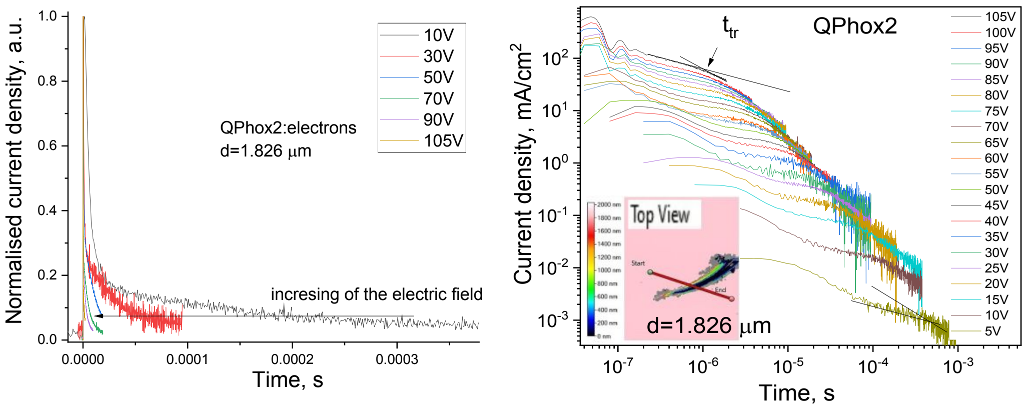

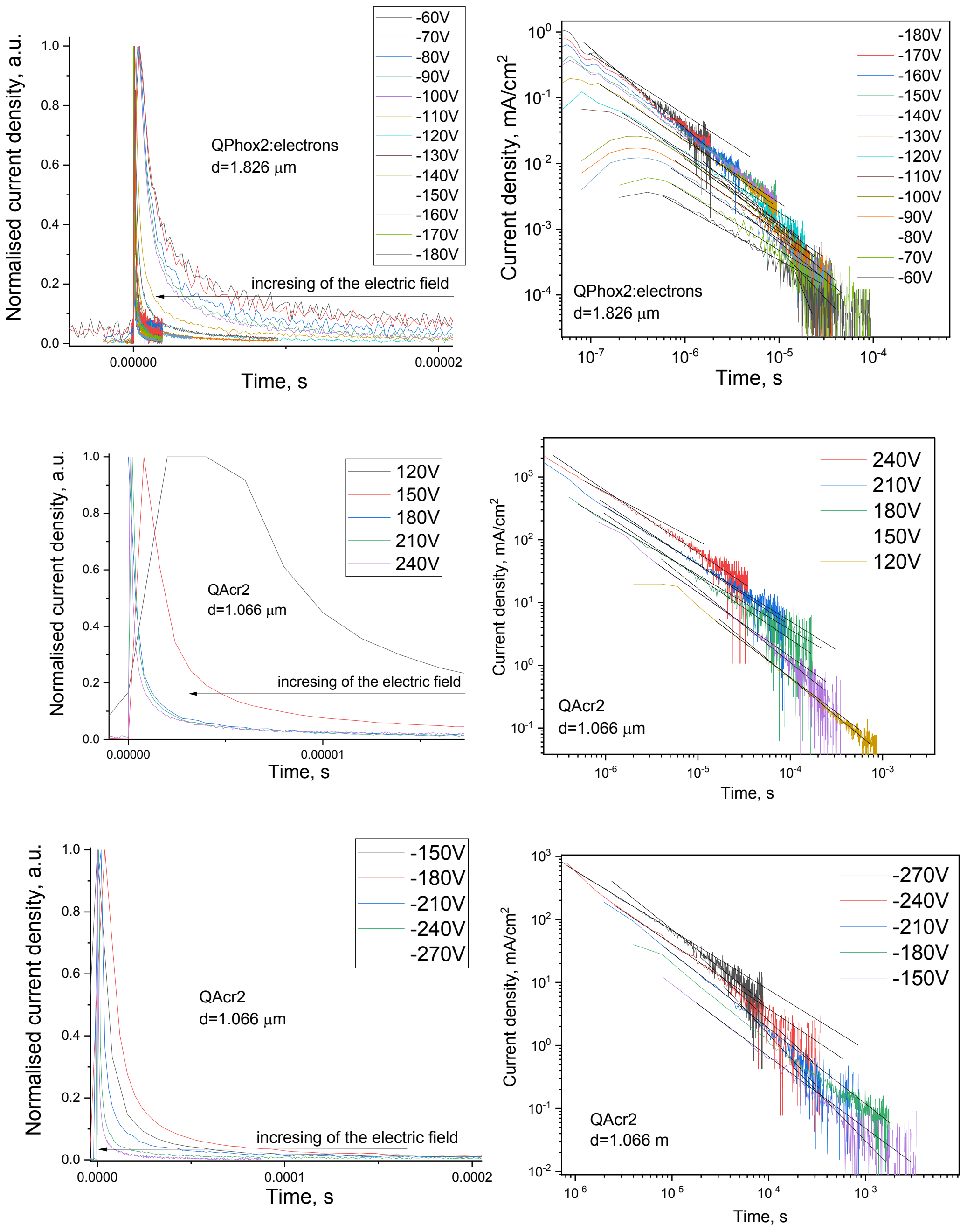

Figure S1. Normalised (left) and non-normalised (right) TOF signals.

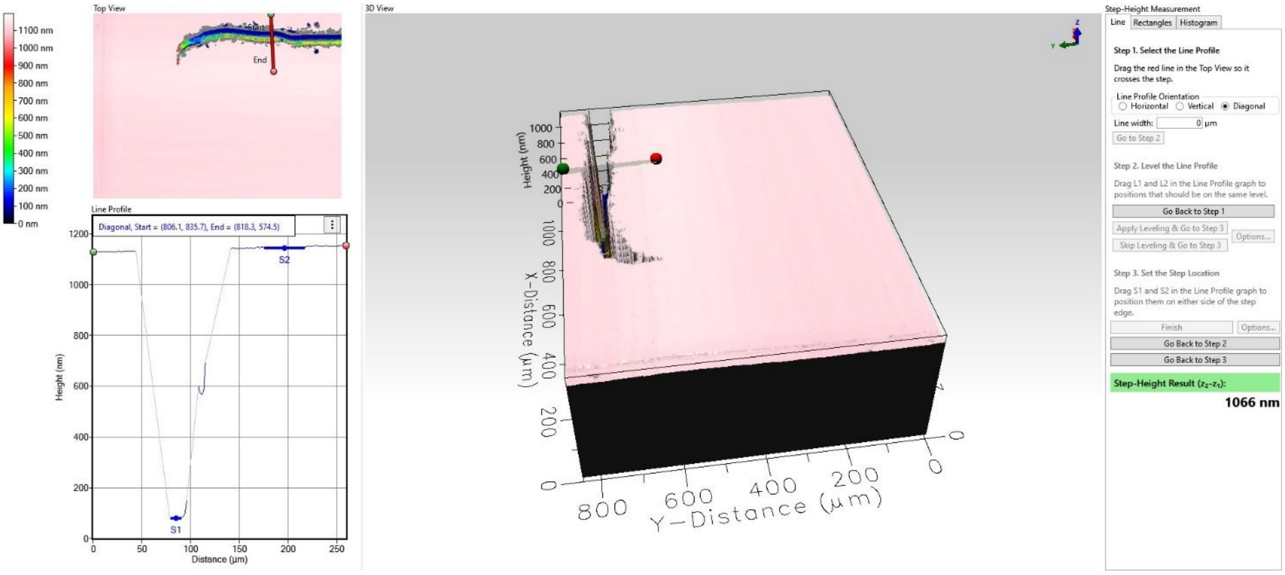

(a)

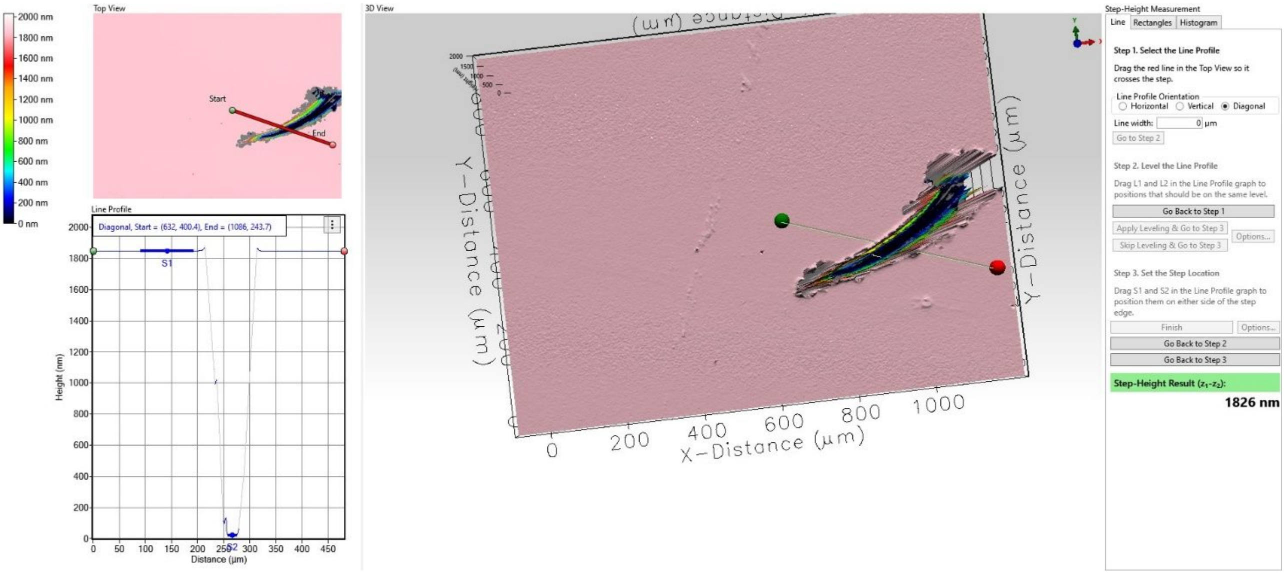

(b)

Figure S2. Thicknesses measurements of TOF samples of compounds **QAcr2** (a) and **QPhox2** (b).

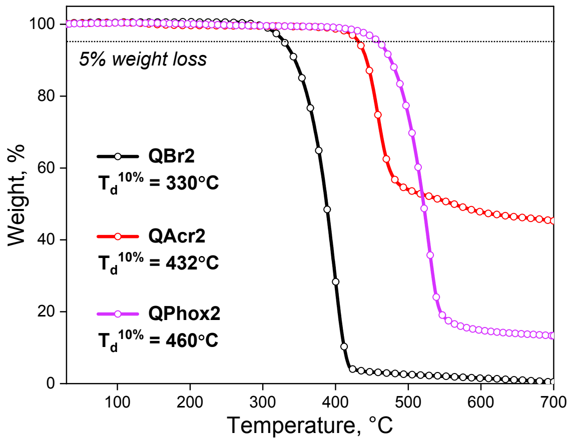

a)

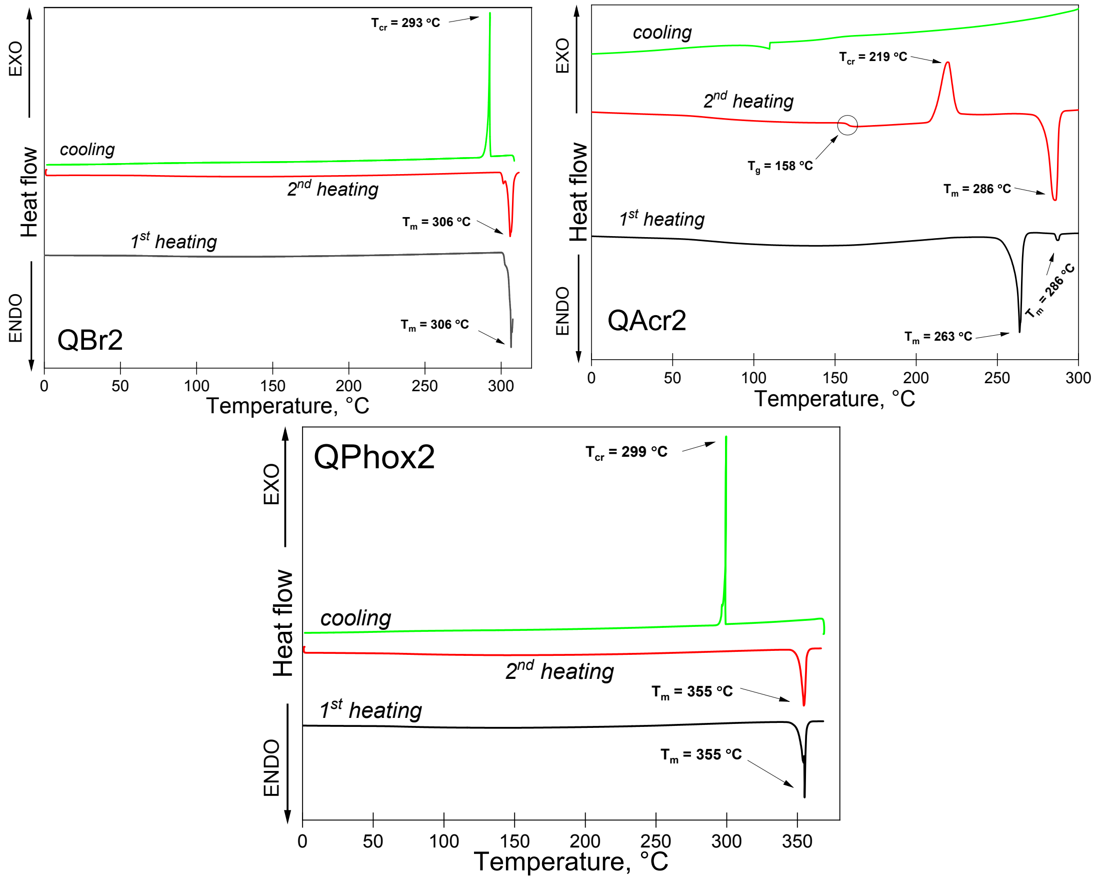

Figure S3. TGA (a) and DSC (b) curves of compounds **QBr2**, **QAcr2** and **QPhox2**.

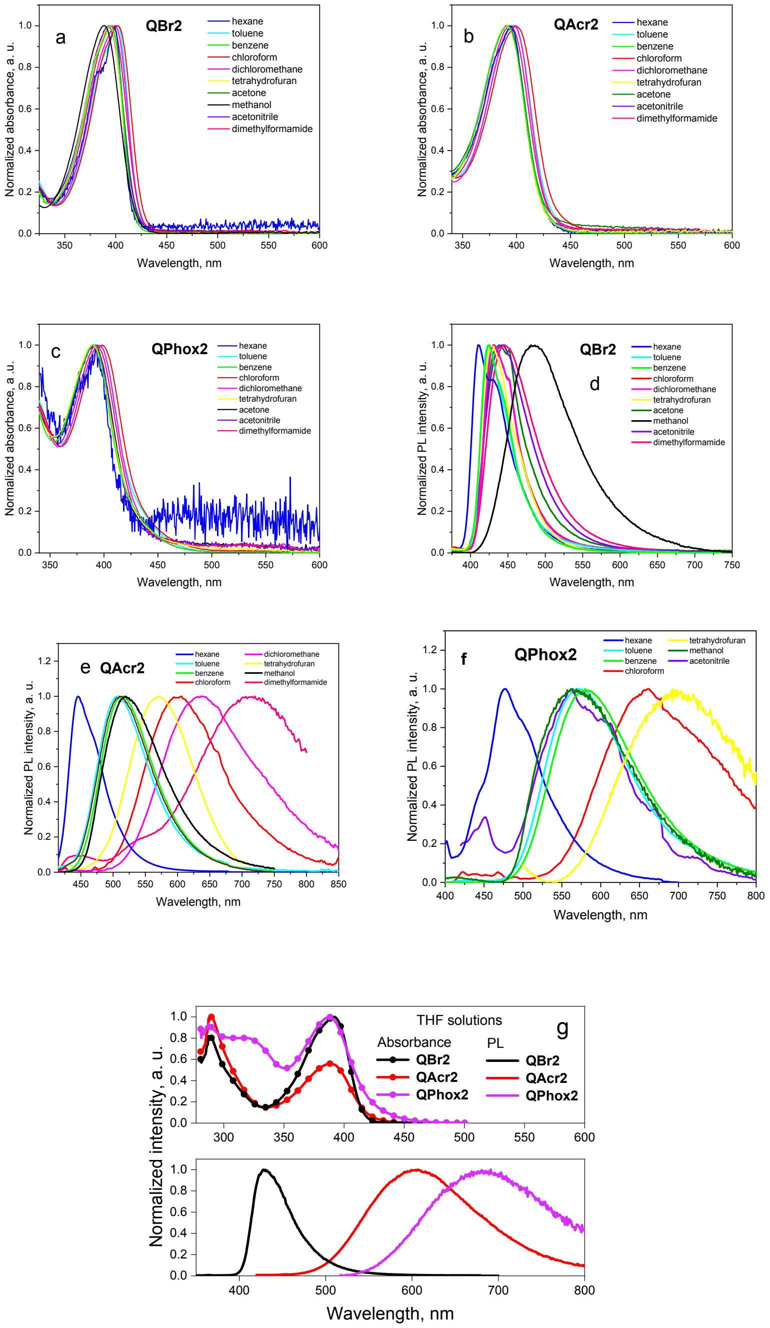

Figure S4. Absorption (a, b, c) and photoluminescence (d, e, f) spectra of different solutions of compounds **QBr2**, **QAcr2**, **QPhox2**. Comparison of absorption and PL (g) spectra of THF solutions of compounds **QBr2**, **QAcr2**, **QPhox2**.

Table S2. Photophysical parameters of solutions of compounds **QBr2, QAcr2,** and **QPhox2**.

Compound	Solvent	λ_{abs}, nm	ε, M^{-1} cm^{-1}	λ_{em}, nm	Δv, cm^{-1}
QAcr2	Toluene	390	84439	513	6147
	Acetone	392	23936	662	10404
	DCM	397	44849	638	9514
	THF	387	106730	613	9526
	Chloroform	400	26577	606	8498
	Acetonitrile	390	15943	544	7258
	DMF	393	39289	720	11556
	Benzene	391	110526	515	6157
	Methanol	397	41531	519	5921
QBr2	Toluene	392	88041	425	1980
	Acetone	393	47962	438	2614
	DCM	400	36932	430	1744
	THF	380	120366	430	3059
	Chloroform	401	25224	432	1789
	Acetonitrile	392	11243	442	2885
	DMF	395	37133	444	2793
	Benzene	395	81225	425	1787
	Methanol	388	32568	481	4983
QPhox2	Toluene	388	71607	570	8229
	Acetone	390	22580	662	10535
	DCM	394	44644	672	10499
	THF	377	102351	693	12095
	Chloroform	398	48331	662	10019
	Acetonitrile	389	5905	564	7976
	DMF	393	22609	444	2922
	Benzene	391	98000	580	8334

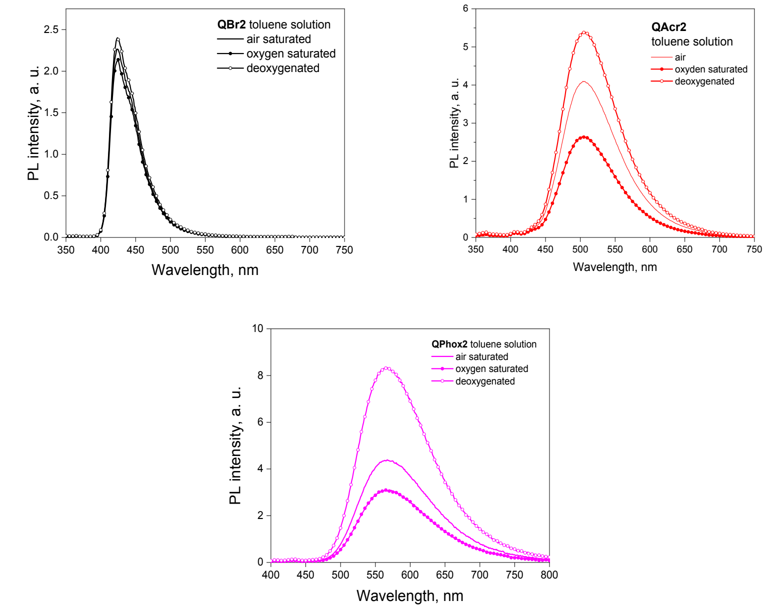

(a)

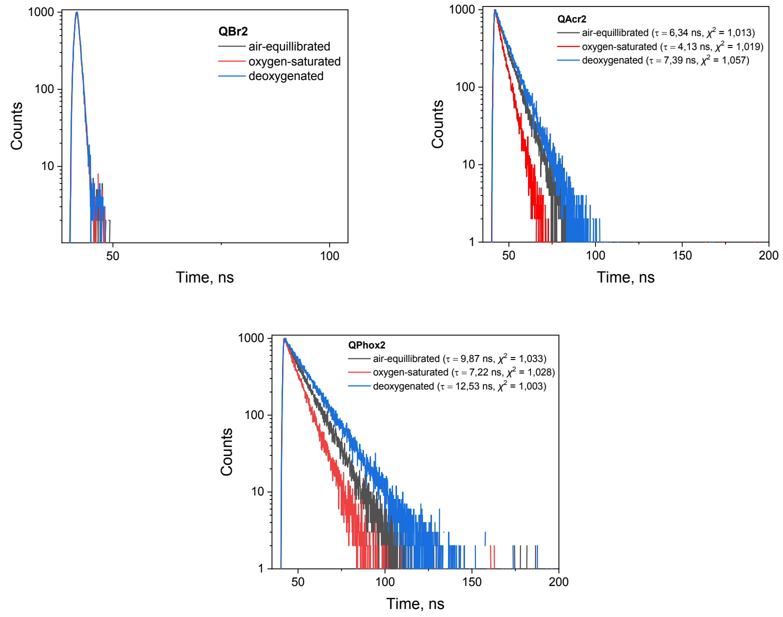

(b)

Figure S5. PL spectra (a) and PL decay curves (b) of compounds **QBr2**, **QAcr2**, **QPhox2** in air equilibrated, oxygen saturated and deoxygenated toluene solutions.

Table S3. Photophysical parameters of **QBr2**, **QAcr2**, **QPhox2**.

Parameter	Sample	QBr2	QAcr2	QPhox2
**λ_{abs}^{max}, nm**	Toluene solution	288, 402	290, 398	288, 330, 396
**λ_{abs}^{max}, nm**	THF solution	288, 391	289, 388	289, 321, 388
**λ_{PL}^{max}, nm**	Toluene solution	427	486	570
**λ_{PL}^{max}, nm**	THF solution	430	602	404, 682
**λ_{abs}^{max}, nm**	Neat film	235, 272, 382, 414	245, 284, 322, 397	245, 277,323, 396
**λ_{PL}^{max}, nm**	Neat film	470	510	569
**FWHM, nm**	Neat film	98	101	117
**E_{S1}, eV**	THF 77K	3.07	2.89	2.63
**E_{T1}, eV**	THF 77K	2.54	2.55	2.45
**ΔE_{ST}, eV**	THF 77K	0.53	0.34	0.18
**PLQY, %**	Toluene solution	15	15.3	12.6
**PLQY, %**	Neat film	2.8	17	23
**λ_{PL}^{max}, nm**	Zeonex air	436	460	489
**λ_{PL}^{max}, nm**	Zeonex vacuum	436, 550	460, 550	489
**λ_{PL}^{max}, nm**	*m*CBP	433, 545	495	535
**E_{S1}, eV**	*m*CBP 77K	3.03	2.84	2.69
**E_{T1}, eV**	*m*CBP 77K	2.67	2.67	2.61
**ΔE_{ST}, eV**	*m*CBP 77K	0.36	0.17	0.08

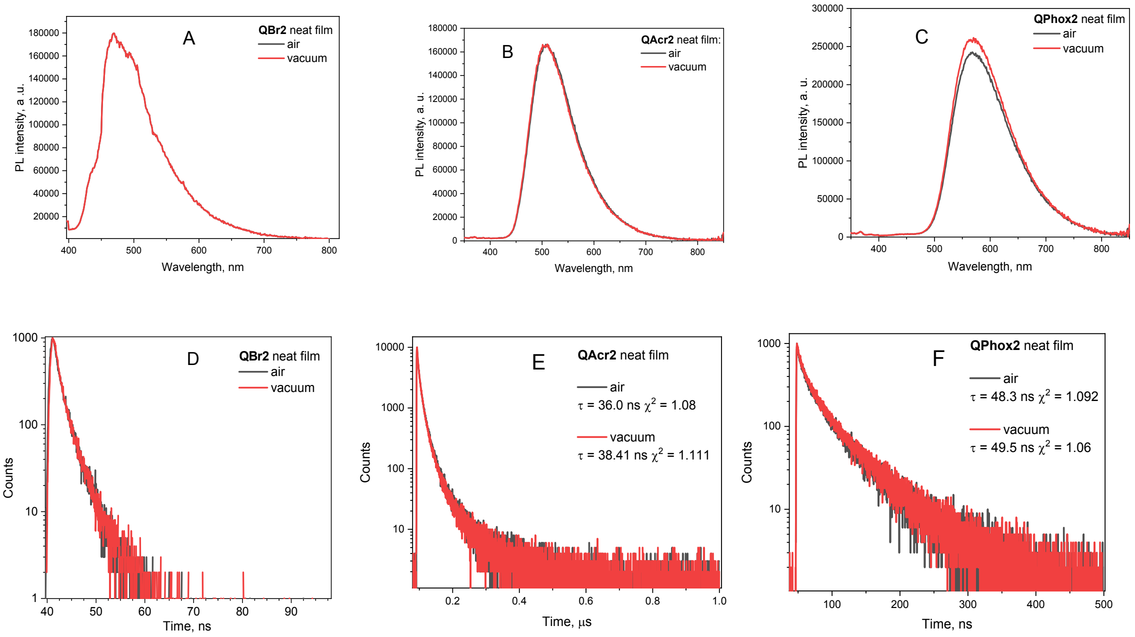

Figure S6. Photoluminescence spectra (a, b, c) and photoluminescence decays (d, e, f) of neat films of compounds **QBr2**, **QAcr2**, **QPhox2** recorded in air and in vacuum.

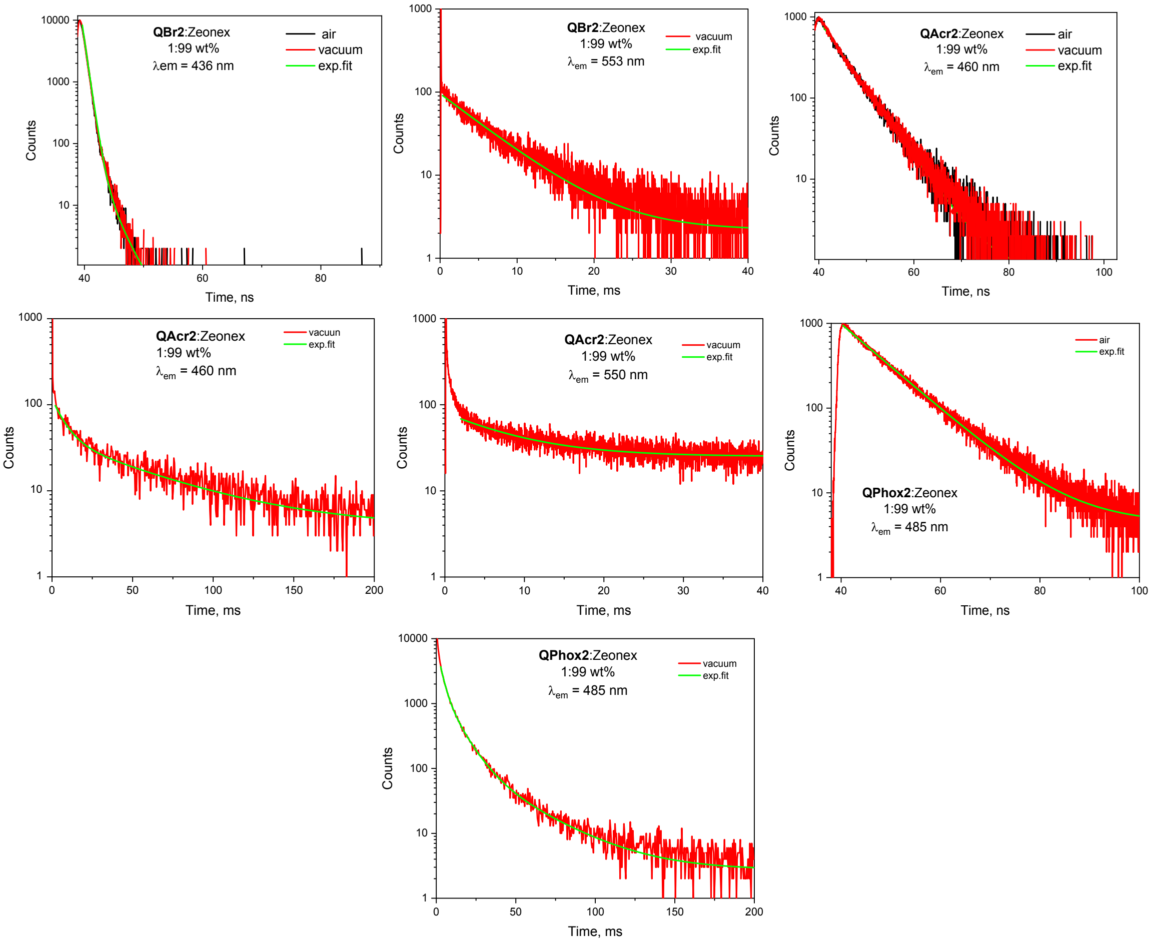

Figure S7. PL decay of 1% of compounds **QBr2**, **QAcr2** and **QPhox2** dispersed in Zeonex, recorded in air and in vacuum.

Table S4. PL decays of 1 wt% of compound **QBr2**, **QAcr2**, **QPhox2** dispersed in Zeonex, recorded in air and vacuum.

Compound	Sample	λ, nm	T	χ²
**QBr2**	Air	436	0,64 ns (94,37 %)	1,241
			1,87 ns (5,63 %)	
	Vacuum	436;	0,65 ns (96,28 %)	1,132
			2,10 ns (3,72 %)	
		553	6105,42 μs (100 %)	1,235
**QAcr2**	Air	460	5,16 ns (100 %)	1,094
	Vacuum	460;	5,17 ns (100%)	1,024;
			7,5 ms (18,84 %)	
			56 ms (81,16%)	1,250
		550	7056,88 μs (100 %)	1,184
**QPhox2**	Air	485	8,4 ns (100%)	1,207
	Vacuum	485	8,6 ns (100%)	1,180
			2,8 ms (29,12 %)	

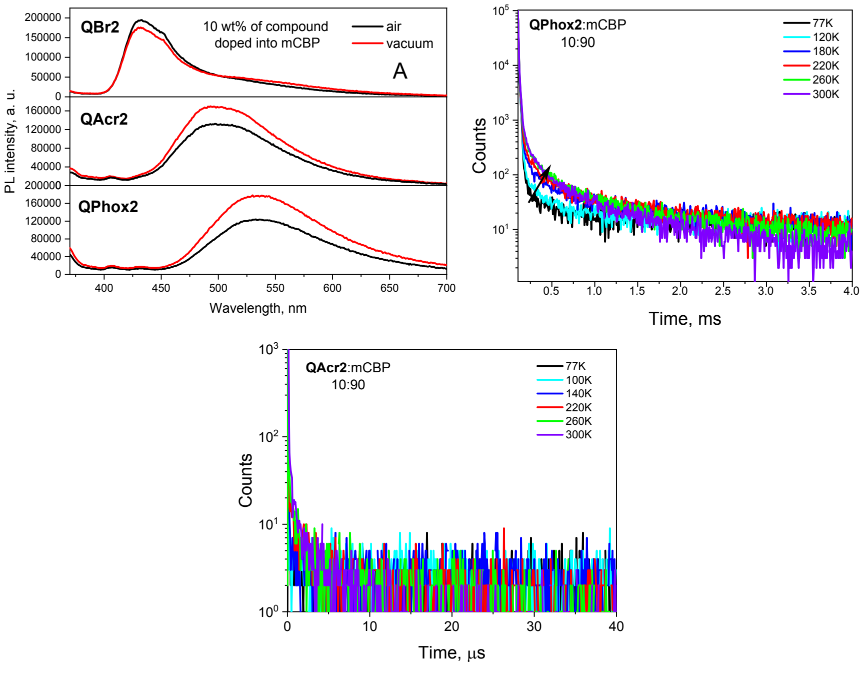

Figure S8. PL spectra of 10 wt% of compound **QAcr2 and QPhox2** dispersed in *m*CBP were recorded at different temperatures.

4. References

(1) Becke, A. D. Density-functional Thermochemistry. III. The Role of Exact Exchange. *J. Chem. Phys.* **1993**, *98* (7), 5648–5652. https://doi.org/10.1063/1.464913.

(2) Lee, C.; Yang, W.; Parr, R. G. Development of the Colle-Salvetti Correlation-Energy Formula into a Functional of the Electron Density. *Phys. Rev. B* **1988**, *37* (2), 785. https://doi.org/10.1103/PhysRevB.37.785.

(3) Ditchfield, R.; Hehre, W. J.; Pople, J. A. Self-Consistent Molecular-Orbital Methods. IX. An Extended Gaussian-Type Basis for Molecular-Orbital Studies of Organic Molecules. *J. Chem. Phys.* **1971**, *54* (2), 724–728. https://doi.org/10.1063/1.1674902.

(4) Frisch, M. J.; Pople, J. A.; Binkley, J. S. Self-consistent Molecular Orbital Methods 25. Supplementary Functions for Gaussian Basis Sets. *J. Chem. Phys.* **1984**, *80* (7), 3265–3269. https://doi.org/10.1063/1.447079.

(5) Yanai, T.; Tew, D. P.; Handy, N. C. A New Hybrid Exchange–Correlation Functional Using the Coulomb-Attenuating Method (CAM-B3LYP). *Chem. Phys. Lett.* **2004**, *393* (1–3), 51–57. https://doi.org/10.1016/J.CPLETT.2004.06.011.

(6) Hirata, S.; Head-Gordon, M. Time-Dependent Density Functional Theory within the Tamm–Dancoff Approximation. *Chem. Phys. Lett.* **1999**, *314* (3–4), 291–299. https://doi.org/10.1016/S0009-2614(99)01149-5.

(7) Tomasi, J.; Mennucci, B.; Cammi, R. Quantum Mechanical Continuum Solvation Models. *Chem. Rev.* **2005**, *105* (8), 2999–3093. https://doi.org/10.1021/CR9904009/ASSET/IMAGES/MEDIUM/CR9904009E00092.GIF.

(8) Vydrov, O. A.; Scuseria, G. E. Assessment of a Long-Range Corrected Hybrid Functional. *J. Chem. Phys.* **2006**, *125* (23), 234109. https://doi.org/10.1063/1.2409292/186324.

(9) Clark, T.; Chandrasekhar, J.; Spitznagel, G. W.; Schleyer, P. V. R. Efficient Diffuse Function-Augmented Basis Sets for Anion Calculations. III. The 3-21+G Basis Set for First-Row Elements, Li–F. *J. Comput. Chem.* **1983**, *4* (3), 294–301. https://doi.org/10.1002/JCC.540040303.

(10) Samanta, P. K.; Kim, D.; Coropceanu, V.; Brédas, J. L. Up-Conversion Intersystem Crossing Rates in Organic Emitters for Thermally Activated Delayed Fluorescence: Impact of the Nature of Singlet vs Triplet Excited States. *J. Am. Chem. Soc.* **2017**, *139* (11), 4042–4051. https://doi.org/10.1021/JACS.6B12124.

(11) Gao, Y.; Geng, Y.; Wu, Y.; Zhang, M.; Su, Z. M. Investigation on the Effect of Connected Bridge on Thermally Activated Delayed Fluorescence Property for DCBPy Emitter. *Dye. Pigment.* **2017**, *145*, 277–284. https://doi.org/10.1016/J.DYEPIG.2017.04.001.

(12) Gaussian 16 Rev. C.01/C.02 Release Notes | Gaussian.com https://gaussian.com/relnotes/ (accessed Dec 8, 2023).

(13) Wang, F.; Ziegler, T. A Simplified Relativistic Time-Dependent Density-Functional Theory Formalism for the Calculations of Excitation Energies Including Spin-Orbit Coupling Effect. *J. Chem. Phys.* **2005**, *123* (15), 154102. https://doi.org/10.1063/1.2061187/347662.

(14) Adamo, C.; Barone, V. Toward Reliable Density Functional Methods without Adjustable Parameters: The PBE0 Model. *J. Chem. Phys.* **1999**, *110* (13), 6158–6170. https://doi.org/10.1063/1.478522.

(15) Ernzerhof, M.; Scuseria, G. E. Assessment of the Perdew–Burke–Ernzerhof Exchange-Correlation Functional. *J. Chem. Phys.* **1999**, *110* (11), 5029–5036. https://doi.org/10.1063/1.478401.

(16) Van Lenthe, E.; Baerends, E. J. Optimized Slater-Type Basis Sets for the Elements 1–118. *J. Comput. Chem.* **2003**, *24* (9), 1142–1156. https://doi.org/10.1002/JCC.10255.

(17) te Velde, G.; Bickelhaupt, F. M.; Baerends, E. J.; Fonseca Guerra, C.; van Gisbergen, S. J. A.; Snijders, J. G.; Ziegler, T. Chemistry with ADF. *J. Comput. Chem.* **2001**, *22* (9), 931–967. https://doi.org/10.1002/JCC.1056.

(18) Marcus, R. A. Electron Transfer Reactions in Chemistry: Theory and Experiment (Nobel Lecture). *Angew. Chemie Int. Ed. English* **1993**, *32* (8), 1111–1121. https://doi.org/10.1002/ANIE.199311113.
